# DNA Fingerprint Analysis of Raman Spectra Captures Global Genomic Alterations in Imatinib-Resistant Chronic Myeloid Leukemia: A Potential Single Assay for Screening Imatinib Resistance

**DOI:** 10.3390/cells10102506

**Published:** 2021-09-22

**Authors:** Rahul Mojidra, Arti Hole, Keita Iwasaki, Hemanth Noothalapati, Tatsuyuki Yamamoto, Murali Krishna C, Rukmini Govekar

**Affiliations:** 1 Advanced Centre for Treatment, Research and Education in Cancer, Tata Memorial Centre, Navi Mumbai 410210, India; rmojidra@actrec.gov.in (R.M.); ahole12345@gmail.com (A.H.); 2 Homi Bhabha National Institute, BARC Training School Complex, Mumbai 400094, India; 3 The United Graduate School of Agricultural Sciences, Tottori University, Tottori 680-8550, Japan; d17a3003@matsu.shimane-u.ac.jp; 4 Faculty of Life and Environmental Sciences, Shimane University, Matsue 690-8504, Japan; nvhnag@life.shimane-u.ac.jp; 5 Raman Project Center for Medical and Biological Applications, Shimane University, Matsue 690-8504, Japan

**Keywords:** chronic myeloid leukemia, array comparative genomic hybridization, Raman spectroscopy, resistance screening, MCR analysis

## Abstract

Monitoring the development of resistance to the tyrosine kinase inhibitor (TKI) imatinib in chronic myeloid leukemia (CML) patients in the initial chronic phase (CP) is crucial for limiting the progression of unresponsive patients to terminal phase of blast crisis (BC). This study for the first time demonstrates the potential of Raman spectroscopy to sense the resistant phenotype. Currently recommended resistance screening strategy include detection of BCR-ABL1 transcripts, kinase domain mutations, complex chromosomal abnormalities and BCR-ABL1 gene amplification. The techniques used for these tests are expensive, technologically demanding and have limited availability in resource-poor countries. In India, this could be a reason for more patients reporting to clinics with advanced disease. A single method which can identify resistant cells irrespective of the underlying mechanism would be a practical screening strategy. During our analysis of imatinib-sensitive and -resistant K562 cells, by array comparative genomic hybridization (aCGH), copy number variations specific to resistant cells were detected. aCGH is technologically demanding, expensive and therefore not suitable to serve as a single economic test. We therefore explored whether DNA finger-print analysis of Raman hyperspectral data could capture these alterations in the genome, and demonstrated that it could indeed segregate imatinib-sensitive and -resistant cells. Raman spectroscopy, due to availability of portable instruments, ease of spectrum acquisition and possibility of centralized analysis of transmitted data, qualifies as a preliminary screening tool in resource-poor countries for imatinib resistance in CML. This study provides a proof of principle for a single assay for monitoring resistance to imatinib, available for scrutiny in clinics.

## 1. Introduction

Targeted therapy of CML with imatinib, a TKI, is the most successful oncotherapy so far [1]. Success of this therapy is, however, limited to the CP, wherein 90% of patients reach hematological remission [2]. In the remaining patients, treatment failure occurs initially (primary resistance), or the initial responders may develop resistance during the course of treatment (secondary resistance) [3]. If the resistant patients are unresponsive to other TKIs, they will progress to the terminal phase of BC, where the survival is 7–11 months [4,5]. Prediction of resistance before or during treatment is therefore necessary to improve the treatment outcome.

In CML, the detection of resistance before and during treatment is performed as per the European Leukemia Network (ELN) protocol [6]. It is recommended that the treatment should be managed in cooperation with a specialized referral center with rapid access to quality-controlled reliable tests for detection of resistance and its molecular basis. Results of this battery of tests also guide the choice of therapy. The tests include qRT-PCR to detect residual disease and assessment of mechanisms of resistance by chromosome banding analysis for additional chromosomal aberrations (CAs), fluorescence in situ hybridization for BCR-ABL1 gene amplification and mutation analysis (Sanger or next-generation sequencing) to detect kinase domain mutations. These tests require specialized technologies, trained manpower and high recurrent cost of consumables. Due to financial and logistical reasons, in resource-poor countries such as India, there are deviations from the ELN protocol. The resultant irregular or lack of screening for resistance [7] may be the reason for the higher number of patients reporting to clinics with advanced disease in India (99% with intermediate to high sokal score) as compared to those in the developed countries (66%) [8]. Further, mechanisms of resistance other than the ones tested, have been reported in BC [9]. This implies the need for a single assay, which is technologically less demanding and relatively inexpensive, as an alternative to the existing battery of tests for screening of resistance, irrespective of the underlying mechanism.

Ours is the first report where we provide evidence for a biophysical tool, Raman spectroscopy, to serve as a single assay for detection of resistance as opposed to the battery of biochemical and molecular biology methods currently used. Chromosomal gains detected by aCGH, in imatinib-resistant CML-BC cell line K562, were captured by multivariate curve resolution analysis (MCR) of the molecular finger-print region of Raman spectra [10], to segregate resistant cells from sensitive cells. Raman spectroscopy is a vibrational spectroscopy and has been shown to be sensitive to composition through molecular finger-print, which has found applications in biology and medicine [11,12,13,14,15,16,17,18]. Raman spectroscopy has a negligible recurrent consumable cost, and low-cost transportable versions are available to serve at peripheral centers [19]. Therefore, if alterations in the DNA content caused by gains and losses are captured by the finger-print region of Raman spectra, it could serve as an inexpensive screen for resistant cells. Advantages and limitations of the observations have been discussed in the light of their merits and demerits in clinical utility.

## 2. Material and Methods

### 2.1. Development of Imatinib-Resistant Cells

The K562 (CML-BC) cell line was gifted by Dr. Tadashi Nagai, Jichi Medical University, Tochigi, Japan. It was maintained in RPMI-1640 medium (Gibco-Life technologies, NY, USA: cat. no. 23400-021), supplemented with 10% fetal bovine serum (Gibco-Life technologies, NY, USA: cat no. 10270-106) and 1% antibiotic (Gibco-Life technologies, NY, USA: cat no. 15240-062). The parent cell line was considered to be sensitive to imatinib (K562S) and its resistant counterpart (K562R) was developed by gradual dose escalation of imatinib (Cell signaling technology, Danvers, MA, USA: cat no. 9084-S) from 0.1 to 0.75 µM and maintained under a constant drug pressure of 0.75 µM thereafter. Development of resistance was confirmed by the MTT assay. In the MTT assay, each well of a 96-well plate was seeded with 7500 cells/100 µL culture media. Upon overnight incubation at 37 °C, the cells were treated with 0.1, 0.5, 1, 5 or 10 µM imatinib for 48 h. DMSO served as the vehicle control. After incubation with DMSO or imatinib, MTT reagent (HiMedia Laboratories, Mumbai, India:TC191) was added to a final concentration of 1 mg/mL per well, incubated for 4–6 h at 37 °C, followed by overnight incubation in 100 µL acidified SDS (10% SDS with 0.01N HCl). Absorbance at 570 nm was recorded, and the IC_50_ value (the concentration which inhibits the survival of 50% of cells) was derived from the plot of % viability versus imatinib concentration.

### 2.2. Array Comparative Genomic Hybridization Analysis

DNA was extracted from K562S and K562R cells with the AllPrep DNA/RNA/miRNA Universal Kit (Qiagen, Germantown, MD, USA: cat no. 80224) according to the manufacturer’s recommendations. Concentration and purity of the extracted DNA were measured with the NanoDrop ND1000 spectrophotometer (Thermo Fisher Scientific, Waltham, MA, USA). The extracted DNA was subjected to aCGH analysis. Briefly, 500 ng of DNA from each test group was labeled by random priming using the SureTag complete DNA labeling kit (Agilent Technologies, Santa Clara, CA, USA: part no. 5190-3400) with Cyanine-5 (Cy-5) fluorescent dye. Reference human DNA from the labeling kit was also labeled simultaneously with Cyanine-3 (Cy-3) dye. Cy-5-labeled DNA from samples and Cy-3-labeled DNA from the reference were quantified, equal quantities were mixed together and hybridized on the SurePrint G3 Human CGH 8 × 60 K microarray (Agilent Technologies, Santa Clara, CA, USA: part no. G4450A) at 67 °C for 24 h. Array images were then acquired using an Agilent laser scanner. Image files were quantified using Agilent’s feature extraction software and analyzed with the Agilent Cytogenomics software, version 2. The ADM-2 algorithm was used, which estimates the standard deviation of the mean log ratio of intervals using the quality-weighted interval score algorithm to compute the aberrations with high precision. The chromosomal gains and losses at distinct regions in K562S and K562R were catalogued and the differentials were identified. The change in amount of DNA in K562R cells was quantified based on the length of CA.

### 2.3. Raman Spectroscopy and Data Analysis

K562S and K562R cell lines (3 × 10^6^ cells) were washed thrice in buffered saline and fixed in paraformaldehyde. Spectra of the cell pellets were recorded using a commercial Raman microscope (Witec alpha300R, Ulm, Germany) and the acquisition parameters were λx 532 nm, 8 mw, 1200 grooves/mm, 10 s acquisition and 10 integrations, cm^−1^. Three technical replicates each, from three biological replicates (9 pellets) of both K562S and K562R, were assessed. Typically, at different positions on the 9 cell pellets, 10 spectra were collected. Preprocessed spectra were baseline-corrected, interpolated and vector-normalized before further analysis. Principal component analysis (PCA) and principal component-linear discriminant analysis (PC-LDA) were carried out by Commercial Unscrambler^®^ X software.

### 2.4. Multivariate Curve Resolution Analysis

MCR analysis was performed using an indigenously developed program specifically for Raman spectroscopic applications in Python [20,21,22], as detailed earlier [10]. Briefly, in MCR, a low-rank approximation of matrix A is obtained by solving the following Equation (1):

A = WH(1)

in which A is an *m* × *n* non-negative Raman hyperspectral data matrix. All elements of W (*m* × *k* matrix), which represents spectral components, and H (*k* × *n* matrix), which represents corresponding abundance profiles, are restricted to be non-negative. Parameter *k* represents the number of spectral components and was set to 5 in this study based on the results of PCA. W and H were iteratively calculated to refine the quality of approximation using alternating least squares so that the Frobenius norm ||A − WH||^2^ was minimized with non-negative constraints W ≥ 0 and H ≥ 0. To obtain sparser solutions, we applied additional penalty terms such as L1-norm (lasso regression) of α^2^ = 0.0015 and L2-norm (ridge regression) of β^2^ = 0.0015, as Equations (2) and (3):

(W^T^W + α^2^E)H = W^T^A (2)

(HH^T^ + β^2^I)W = HA^T^ (3)

where E is a *k* × *k* matrix whose elements are all unity, and I is a *k* × *k* identity matrix.

## 3. Results

### 3.1. IC_50_ Was Ten-Fold Higher in Imatinib-Resistant Cells

IC_50_ is a concentration of the compound under study at which 50% of the cells remain viable. In the present study, IC_50_ for imatinib of parental K562S was found to be 0.75 µM. IC_50_ for K562R cells was found to be more than 10 µM. K562R cells were constantly maintained under 0.75 µM imatinib, IC_50_ for K562S, and cells grew with no loss of viability. The S and R cells thus served as an appropriate model system to study imatinib resistance.

### 3.2. Resistant Cells Showed Increased Chromosomal Gains in Genomic Analysis

**Figure 1:**
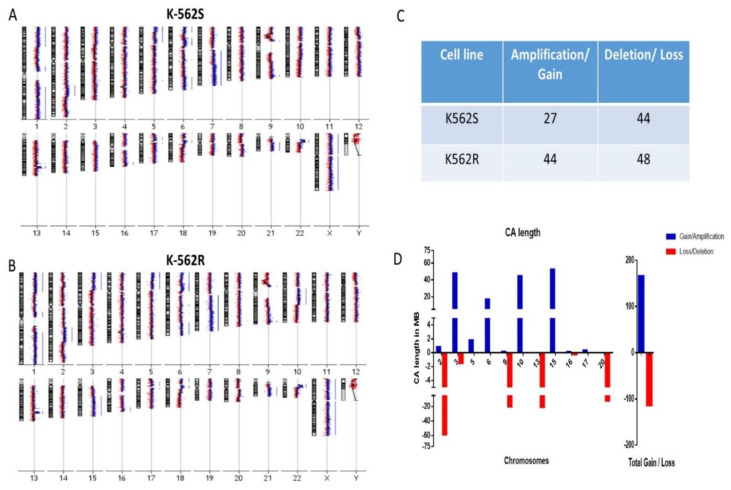
Array CGH profile: (**A**) K562S, (**B**) K562R generated from Agilent Cyto genomics software (blue-amplification/gain, red-deletion/loss). (**C**) Total losses and gains in the K562S and K562R cells. (**D**) Histogram representing the altered DNA content due to chromosomal aberrations differentially present in K562R cells (blue-amplified/gain, red-deletion/loss).

Chromosomal losses and gains were detected in both K562S (Figure 1A) and K562R (Figure 1B), based on the LogR intensities of reference and test DNA in aCGH. In comparison to reference DNA, K562R cells harbored a total of 92 CAs, which included 44 gains and 48 losses, whereas K562S cells showed a total of 71 CAs, with 27 gains and 44 losses (Figure 1C) (Supplementary Reports S1 and S2). The comparative analysis between both cell lines revealed a higher number of CAs (gains and losses) in K562R cells as compared to K562S cells. Based on the length of CAs, change in the content of DNA was calculated and it reflected an increase in the DNA content (52.45 Mb) in K562R cells, as shown in Figure 1D (Supplementary Table S1).

### 3.3. Discrimination of Resistant Cells by Finger-Print Analysis of Raman Spectra

#### 3.3.1. Average Raman Spectrum of Sensitive and Resistant Cells

**Figure 2:**
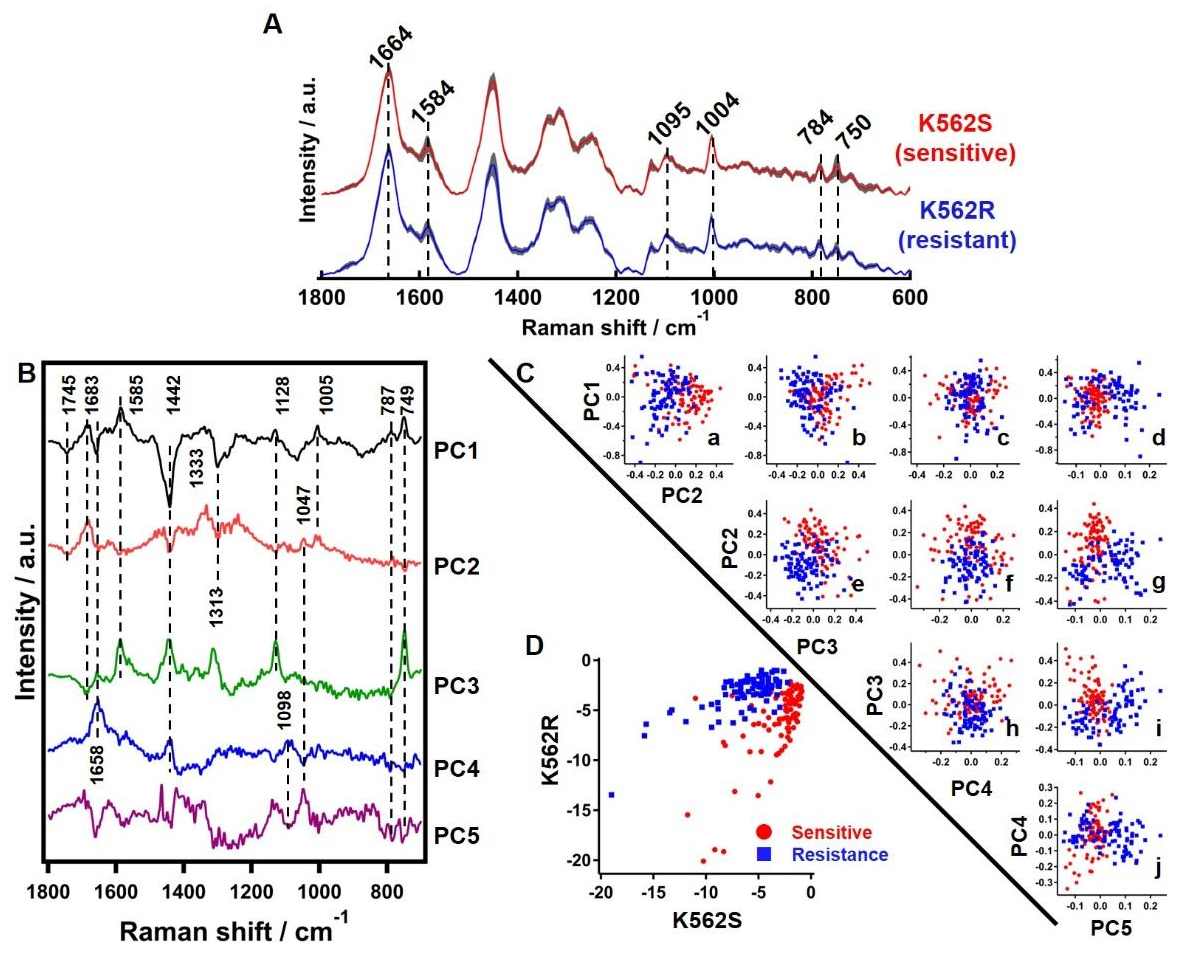
Comparison of average Raman spectra, PCA and LDA. (**A**) K562S (Red) and K562R (Blue) cell pellets along with their ±standard deviation (Grey). Some of the prominent bands observed in both are highlighted using dashed lines. (**B**) Discrimination of K562S and K562R cells by PCA. First 5 PC loadings’ spectra indicting molecular information. Contribution of each: PC1 (41%), PC2 (20%), PC3 (12%), PC4 (9%) and PC5 (3%). Dashed lines indicate some important bands useful for molecular identification. (**C**) PC scores scatter plot helpful to understand discrimination capability. (a) PC2, (b) PC3, (c) PC4 and (d) PC5 vs. PC1, respectively. (e) PC3, (f) PC4 and (g) PC5 vs. PC2, respectively. (h) PC4 and (i) PC5 vs. PC3, respectively. (j) PC5 vs. PC4. Red circles represent K562S cells and blue boxes represent K562R cells. (**D**) Linear discrimination factors of K562S and K562R cells are plotted by red circles and blue boxes, respectively.

Averages of Raman spectra obtained from 90 points each of K562S and K562R cell pellets are presented in Figure 2A. Prominent Raman bands corresponding to major biomolecules observed in both types of cells include pyrrole ring (750 cm^−1^) and cytochromes (1584 cm^−1^) [23], cytosine and O-P-O symmetric stretch (784 cm^−1^) and PO_2_^−^ stretch (1095 cm^−1^) from DNA [24], phenyl alanine ring breathing from proteins (1004 cm^−1^) and amide I/−C = C− stretch (1664 cm^−1^) from proteins and lipids [25,26]. Though we expected differences in the average spectrum of the two groups of cells, especially for DNA marker bands due to the differences in DNA content demonstrated by aCGH, no significant difference was observed when considering their standard deviations (Figure 2A).

#### 3.3.2. Principle Component Analysis of Raman Hyperspectral Data

PCA identifies major components, called principal components (PCs), and provides their individual contribution to the whole dataset. In this dataset, PCA identified 5 PCs with a total contribution of 85%, and further additions of PCs did not contribute significantly. The outcome of PCA analysis with 5 PCs is presented in Figure 2. Examination of PC loadings (Figure 2B) provides biochemical information. PC1 and PC2 loadings’ spectrum, which are the two largest components, had mixed contributions from proteins (1005 and 1683 cm^−1^), lipids (1442, 1658 and 1745 cm^−1^), cytochromes (749, 1128, 1585 and 1313 cm^−1^) and DNA (784 cm^−1^). It is important to note that Raman bands such as 1745 cm^−1^ and 1683 cm^−1^ of lipids and proteins respectively, which are not clearly visible in average spectra, can be observed in the first two PCs. PC3 is dominated by bands of cytochrome origin (750, 1128, 1313 and 1584 cm^−1^). PC4 and PC5 are primarily composed of lipids and DNA. It is apparent from PCA that biomolecules such as protein, lipids, cytochrome and DNA have maximum contributions to Raman hyperspectral data (Table 1).

The PC scores plot shows that different combinations of PCs could moderately segregate the sensitive and resistant cells. A scattering plot of each of the 5 PC scores with respect to the others is presented in Figure 2C. The plot of PC1 vs. PC2 implies that PC2 may have potential to discriminate K562S and K562R (Figure 2C(a)). In addition to this, we looked into all possible combinations among the first 5 PCs and observed that PC2 vs. PC3 (Figure 2C(e)) and PC2 vs. PC5 (Figure 2C(g)) may also separate the two groups to a fair degree.

#### 3.3.3. Linear Discriminant Analysis of Raman Hyperspectral Data

LDA using the first 5 PCs discussed earlier achieved a good degree of discrimination between the two groups (Figure 2D). Overall accuracy was 93.9%, with correct classification of 92% and 96% of K562S and K562R, respectively.

#### 3.3.4. Multivariate Curve Resolution Analysis of Raman Spectra

**Figure 3:**
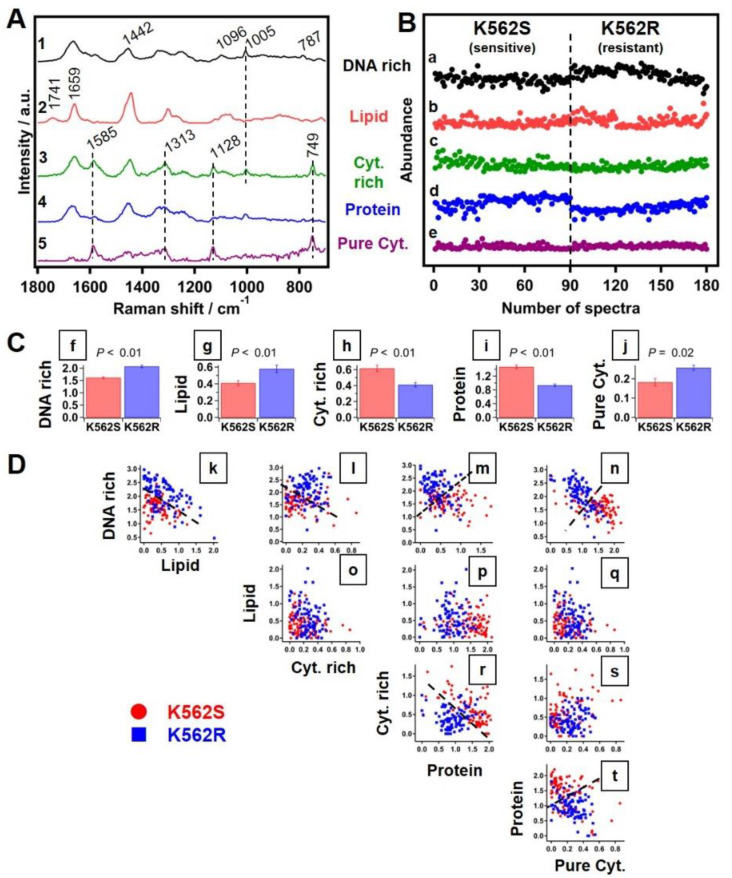
Five components in exploratory MCR analysis. (**A**) MCR-extracted spectral components, (1) DNA-rich, (2) lipids, (3) cytochrome-rich (Cyt. rich), (4) proteins and (5) pure cytochrome (Pure Cyt.). (**B**) Corresponding MCR-extracted abundance profiles (a–e). Broken line in (**B**) separates sensitive and resistant spectral data. (**C**) Average abundance histogram of (f) DNA-rich, (g) lipids, (h) Cyt. rich, (i) proteins and (j) Pure Cyt. of the two cell groups. Error bars are standard error of mean. *p*-values obtained by *t*-test are denoted on top of each histogram. (**D**) Two-dimensional scatter plots of abundances: (k–n) lipid, Cyt. rich, proteins and Pure Cyt. vs. DNA-rich component, (o–q) Cyt. rich, proteins and Pure Cyt. vs. lipid, (r–s) proteins and Pure Cyt. vs. Cyt. rich and (t) protein vs. pure Cyt., respectively. Dashed lines are drawn as visual guides to separate sensitive and resistant cell groups.

MCR analysis to investigate the molecular basis of segregation was performed and the results are presented in Figure 3. The same 5 PCs used in PCA and LDA were used to construct a MCR model. It is important to note that, unlike PCA loadings’ spectra, MCR component spectra contain only positive features, resulting in physically meaningful and interpretable pure Raman spectra, as shown in Figure 3A. Based on the spectral profiles, extracted spectral components were identified to be “DNA-rich” (Figure 3(A1)), “lipid” (Figure 3(A2)), “cytochrome-rich” (Figure 3(A3)), “protein” (Figure 3(A4)) and “pure cytochrome” (Figure 3(A5)), respectively. MCR analysis also provided corresponding abundance profiles for each component, as shown in Figure 3B. As seen in Figure 3B(a), the abundance of the DNA-rich component is different in the two groups. Further clarity on this difference was obtained from average abundance along with standard errors in each group (Figure 3C). Except for the pure cytochrome component, all molecular components showed significant differences between the sensitive and resistant groups. Interestingly, the abundance of DNA (Figure 3C(f)) and lipid (Figure 3C(g)) components seems to be higher in the resistant group, as compared to its sensitive counterpart. On the other hand, cytochrome-rich (Figure 3C(h)) and protein (Figure 3C(i)) components show the opposite trend. Further, construction of two-dimensional scatter plots of abundances of each molecular group versus the other and the results are shown in Figure 3D. The outcome of MCR analysis thus confirmed that that ratio of DNA to all other components is high in resistant cells as compared to the sensitive cells (Figure 3D(k–n)).

## 4. Discussion

Detection of resistance to imatinib as well as the mechanism underlying resistance is crucial for decisions about the choice of therapy for resistant CML, and in turn, treatment outcome. Towards this end, a battery of tests are carried out in clinical laboratories, which are technologically and financially demanding. To cater to the need of resource-poor countries where these tests cannot be performed for majority of patients due to financial and logistical reasons [7], we proposed a single method which has the ability to segregate resistant cells.

CAs are associated with leukemia [27], and there is an increase in CAs with disease progression which co-occurs with the development of resistance [28]. Additionally, in CML patients with advanced disease, unresponsiveness to imatinib increases. Additional CAs such as gains and losses are reported in 10% of CP patients and 16% of imatinib-resistant CP patients, and accumulation of more aberrations is observed in 40–50% of patients in advanced phases [29], and several studies suggest their role in disease progression and resistance [30,31,32]. Earlier, we studied patient samples to identify CAs which could predict secondary resistance and to further delineate the causative role of these aberrations in development of resistance. In order to demonstrate the causative role, we developed a cell model for resistance and profiled it for CAs to discover the overlap with the patient CA profile developed earlier, which was the initiator of the present investigation. We carried out aCGH of CML-BC cell line K562, both sensitive and resistant to imatinib. aCGH is a specialized technique to detect losses and gains in chromosomal regions, with high sensitivity and specificity [33]. We found that the imatinib-resistant cells had a higher number of CAs as compared to the sensitive cells. This corroborated with the observations in patient samples found in our parallel study (data not shown). By simple summation of the lengths of different regions gained and lost, we found an increase in the DNA content (52.45 Mb) in resistant cells as compared to the sensitive cells, as shown in Figure 2D. The increase in DNA content was due to chromosomal gains alone and not due to alterations in ploidy of K562R cells (data not shown). However, aCGH is a technologically demanding and expensive test and our observations were not translatable into a single inexpensive test for resistance, which was what we wanted to develop.

We found an opportunity in the report by Iwasaki et al. [10], on MCR analysis of the molecular finger-print region of Raman spectra to distinguish normal and transformed mammary epithelial cells. Raman spectroscopy is a vibrational spectroscopy and has been shown to be sensitive to cell composition through “molecular finger-print”, which has found applications in biology and medicine [11,13,34,35]. Raman spectroscopy has a negligible recurrent consumable cost, and low-cost transportable versions of the spectroscope are available to serve at peripheral centers [19]. The assay can be performed on intact cells isolated from blood, which is a procedure commonly performed in primary health centers for routine blood tests. Capturing of the spectra of cell pellets is not technically demanding. The captured spectra can be transmitted to central hospitals for diagnosis, thereby spreading the reach of resistance monitoring.

In K562 cells sensitive and resistant to imatinib, we found that the average spectral pattern as well as the DNA marker bands at 784 and 1095 cm^−1^ could not segregate the two cell populations considering their standard deviations (Figure 2A). A simple approach would be to calculate biomolecular ratios using each individual Raman band (univariate approach), but such an analysis would be inconclusive. This is primarily because each Raman band may have contributions from more than one molecular component and information about molecular components could be spread across the spectrum. Therefore, authentic molecular information of each molecular component can be obtained only by assessing the entire Raman spectrum. We employed various multivariate statistical analyses, such as PCA, LDA and MCR analysis, to hyperspectral data to develop a robust screening tool based on Raman spectroscopy.

PCA is a commonly applied multivariate method primarily helpful in dimensionality reduction of large datasets. Essentially, PCA identifies major components, PCs, and provides their individual contribution to the whole dataset. We observed that different PCs fairly contributed to segregation of resistant and sensitive cells. However, PCA cannot extract pure biomolecular spectral components or their abundances. Additionally, with both positive and negative features in their loading spectra, with mixed information from multiple molecular components, one may understand the correlation between some molecular groups, but it would be difficult to interpret in a physically meaningful manner. Similarly, LDA also discriminated the two populations. While PCA-LDA showed high accuracy of discrimination between sensitive and resistant cells, it was still difficult to discuss the factors responsible for discrimination due to the lack of pure molecular information. This particularly affects the translation of such Raman spectroscopy-based techniques for practical screening applications. To overcome this problem, we applied MCR analysis to investigate the molecular basis, and the results were presented in Figure 3.

MCR analysis revealed spectral components which corresponded to DNA-rich, lipid, cytochrome-rich and protein data, and it was observed that the abundance of DNA-rich and lipid components is different in the two groups (Figure 3B(a)). With the information about mean abundances, we further wanted to investigate which of these differences are crucial to discriminate the two cell groups. Since abundance profiles obtained from MCR are not absolute, it would be difficult to use such information to screen cell types. However, relative concentrations are meaningful in the given context and can be quite useful for real applications. Therefore, construction of two-dimensional scatter plots of abundances of each molecular group versus the others was carried out, and the results are shown in Figure 3D. It was finally apparent that the ratio of DNA to all other components is high in the resistant cell group compared to the sensitive type (Figure 3D(k–n)). The abundance of DNA in resistant cells from Raman-MCR analysis corroborates well with the increased DNA content in resistant cells associated with increased CAs observed in the genomic analysis reported in this study. Though other components such as protein seem to have potential to discriminate these cells (Figure 3D(r–t)), it is difficult to depend on the protein Raman component, as it cannot be ascertained to any single particular protein. Therefore, with the higher abundance of DNA in the resistant cell group, we can conclude that the relative ratio of DNA to other molecular components can be used as a reliable marker for the discrimination of resistant from sensitive cells. Once the discrimination model is established, acquisition of Raman spectra and consequent analysis can be performed quickly compared to traditional biochemical and molecular biological methods.

In our exploratory study, Raman spectroscopy combined with MCR analysis segregated sensitive and resistant cells based primarily on abundance of DNA, which corroborates very well with aCGH results. The routinely used biochemical assays for quantifying DNA lack the specificity and sensitivity necessary for precise screening of change in the DNA content due to copy number variations. However, the abundance of DNA in resistant cells can be identified by Raman-MCR analysis, which can further segregate both groups. Therefore, this study serves as a proof of principle for the application of MCR-assisted Raman micro-spectroscopy to capture altered DNA content due to copy number variations in resistant cells under study, which holds merit for its assessment in the clinic as a rapid screening tool for TKI-resistant CML.

While validating the application of this approach in screening of resistance mechanisms in CML, important points need to be addressed. Currently, the screening is based on testing for kinase domain mutations and BCR-ABL gene amplification, which in turn guides the choice of therapy in resistant patients. However, detection of altered levels of imatinib/other transporters of TKIs as well as acquisition of additional chromosomal aberration, which occurs during progression to BC, are not tested. Raman spectra of peripheral blood cells from patients are anticipated to provide a comprehensive picture of resistance, with stratifications which can guide the choice of therapy. The spectral data need to be compared with acquisition of resistance to the first line of treatment and the response to subsequent treatment. The ability of Raman spectra to predict the choice of treatment in resistant patients would endorse its qualification as a single assay in resistance screening of CML.

## Figures and Tables

**Table 1:** List of biomolecular components and their corresponding wavenumbers observed in PCA.

Biomolecules	Wavenumbers
Proteins	1005 and 1683 cm^−1^
Lipids	1442, 1658 and 1745 cm^−1^
Cytochromes	749, 1128, 1313 and 1585 cm^−1^
DNA	784 cm^−1^

## Data Availability

The data presented in this study are available from the corresponding authors upon reasonable request.

## Supplementary Materials

The following are available online at www.mdpi.com/article/10.3390/cells10102506/s1, Report S1: K562-R aCGH profile; Report S2: K562-S aCGH profile; Table S1: K562R DNA content associated with CA.
