# Supplementary material for: DNA Fingerprint Analysis of Raman Spectra Captures Global Genomic Alterations in Imatinib-Resistant Chronic Myeloid Leukemia: A Potential Single Assay for Screening Imatinib Resistance
Source: Cells. 2021 Sep 22;10(10):2506. doi: 10.3390/cells10102506 (PMC8533852; doi:10.3390/cells10102506)
Supplement: Supplementary file 1 [file cells-10-02506-s001.zip › Report S1-K562-R aCGH profile.pdf]

Sample Information

Green Sample	:	Research K562-R
Array ID	:	252192435816_2_3
Global Display Name	:	252192435816_2_3
Polarity	:	-1
Red Sample	:	Reference DNA
DerivativeOfLogRatioSD	:	0.324231
Intermediate Report by	:	DATASYSTEM\admin

Genome View (Amp/Del)

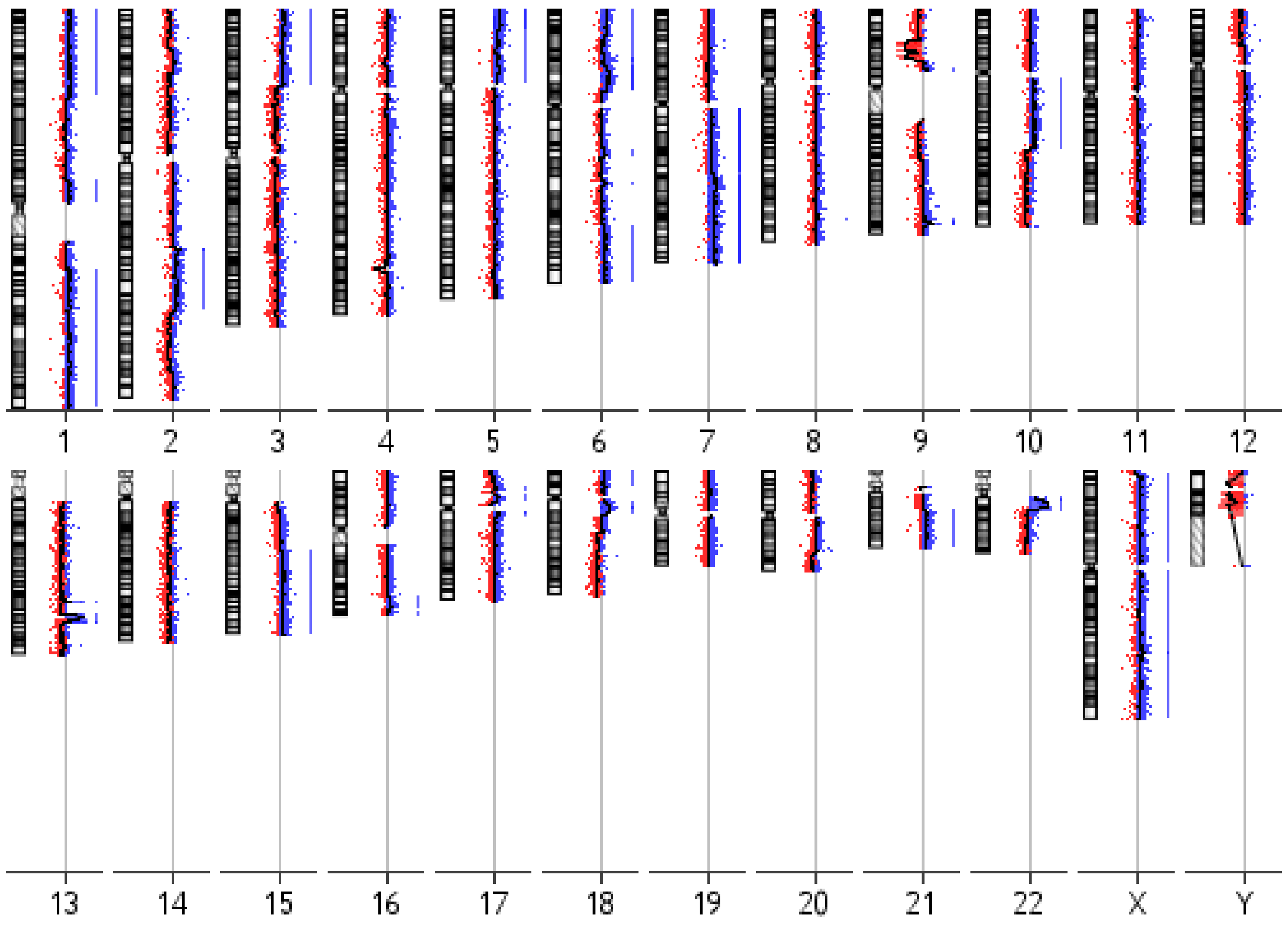

This is an intermediate report and not a final signed off report

Chromosome Views (Amp/Del)

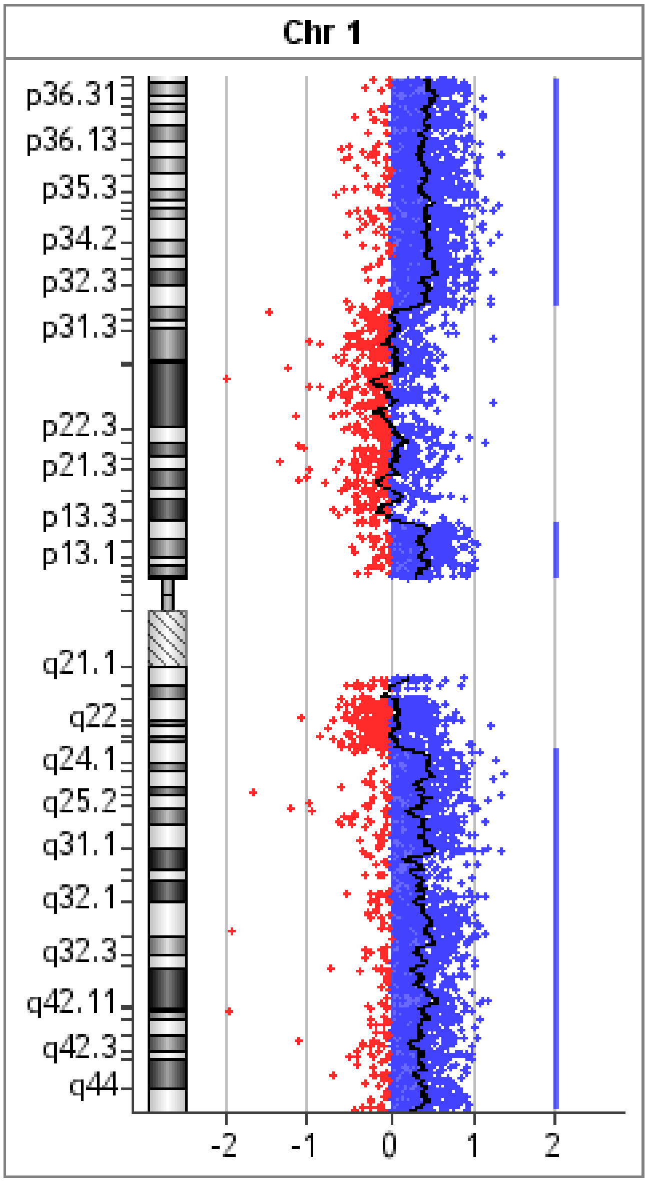

This is an intermediate report and not a final signed off report

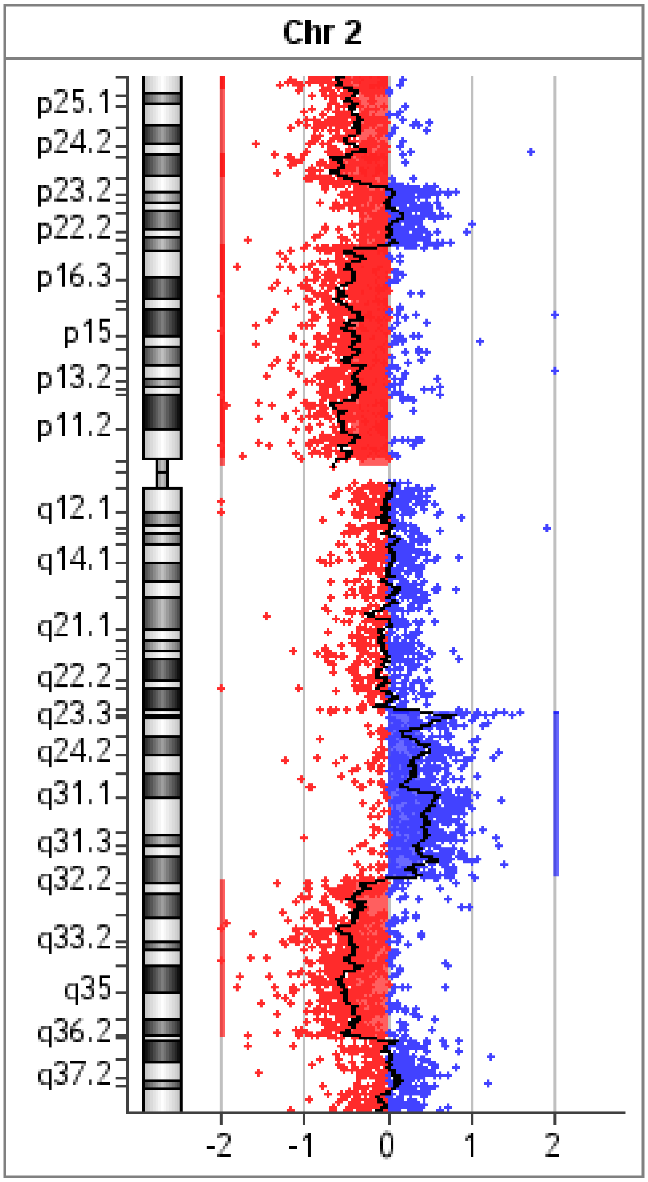

This is an intermediate report and not a final signed off report

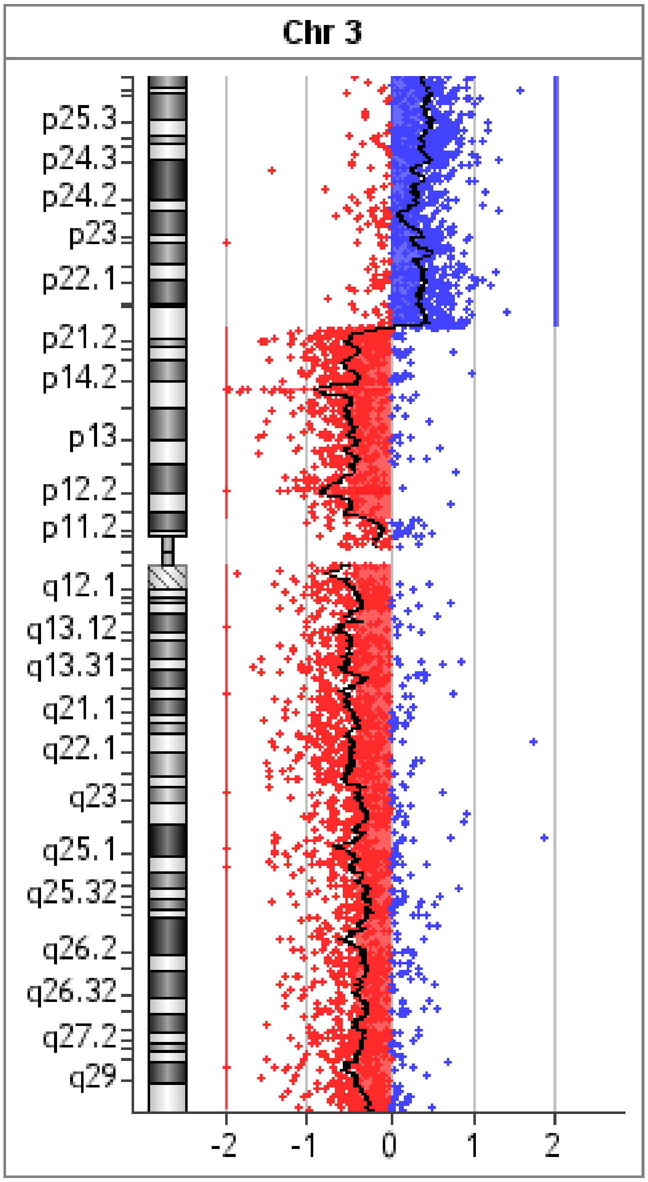

This is an intermediate report and not a final signed off report

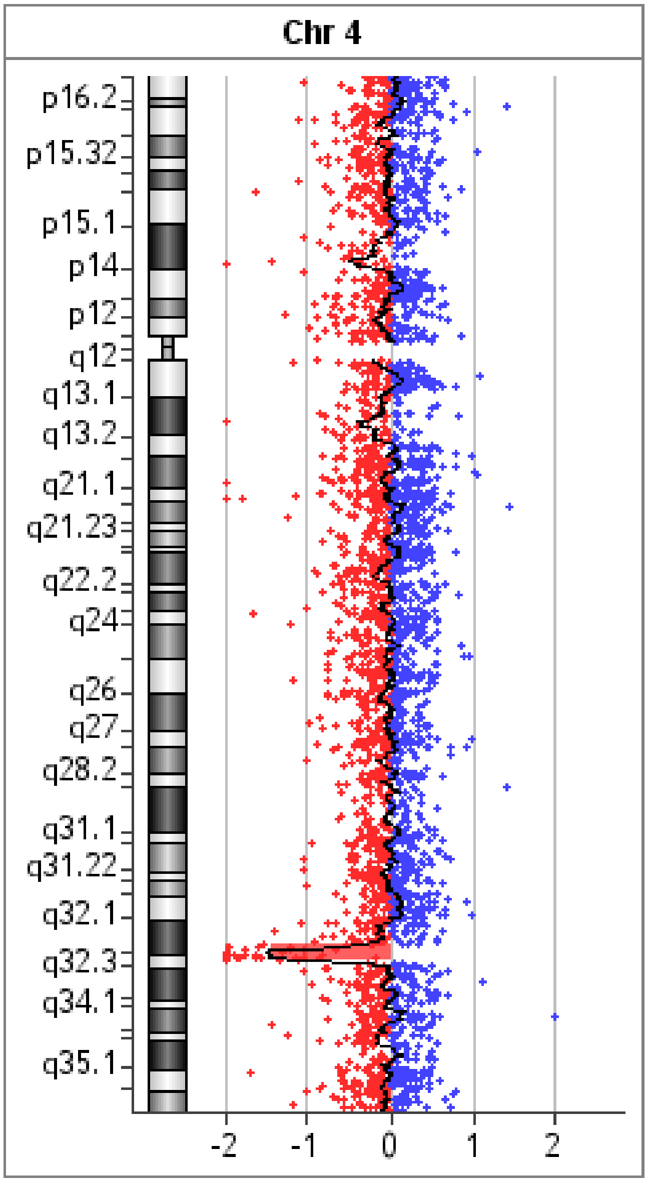

This is an intermediate report and not a final signed off report

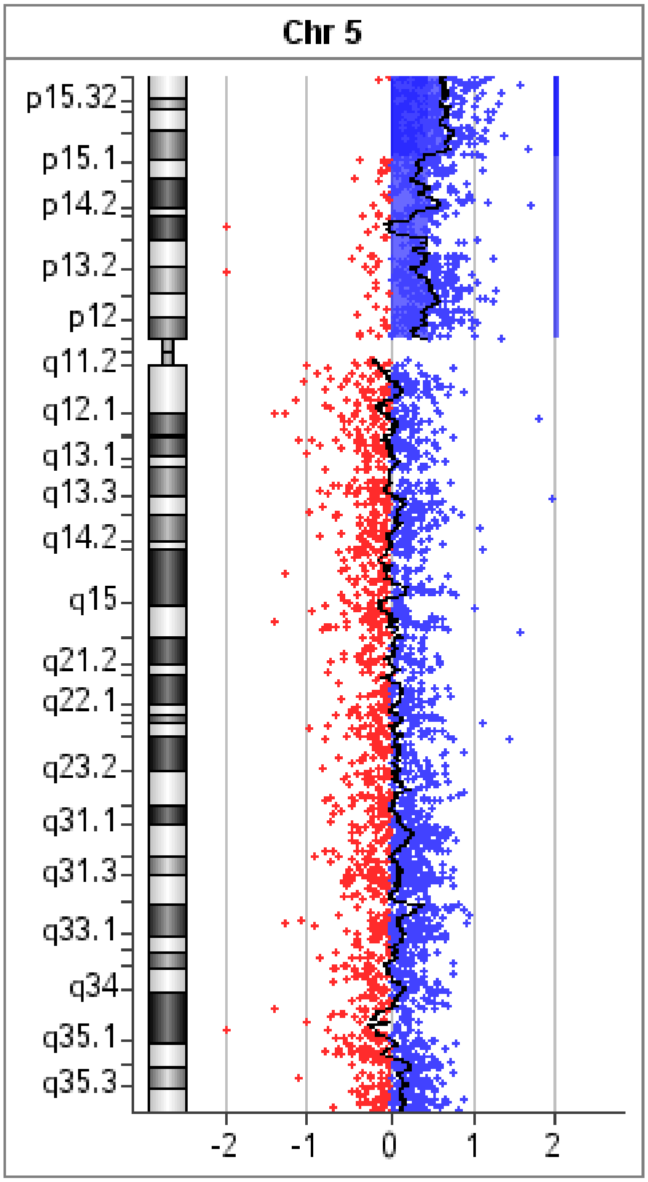

This is an intermediate report and not a final signed off report

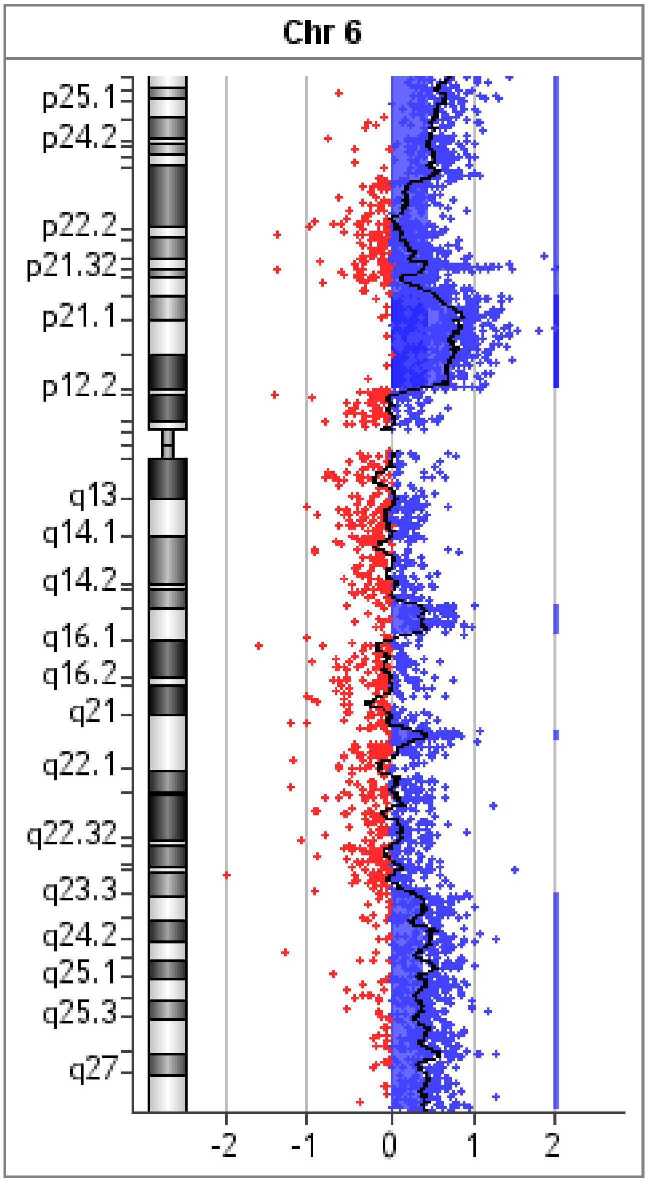

This is an intermediate report and not a final signed off report

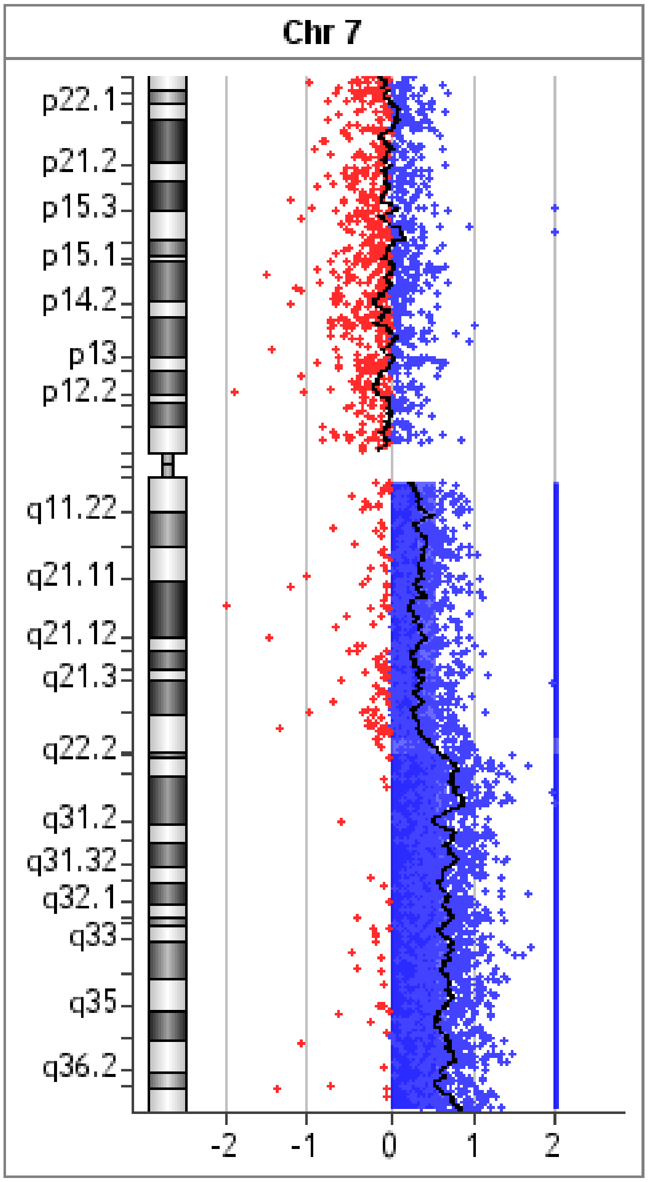

This is an intermediate report and not a final signed off report

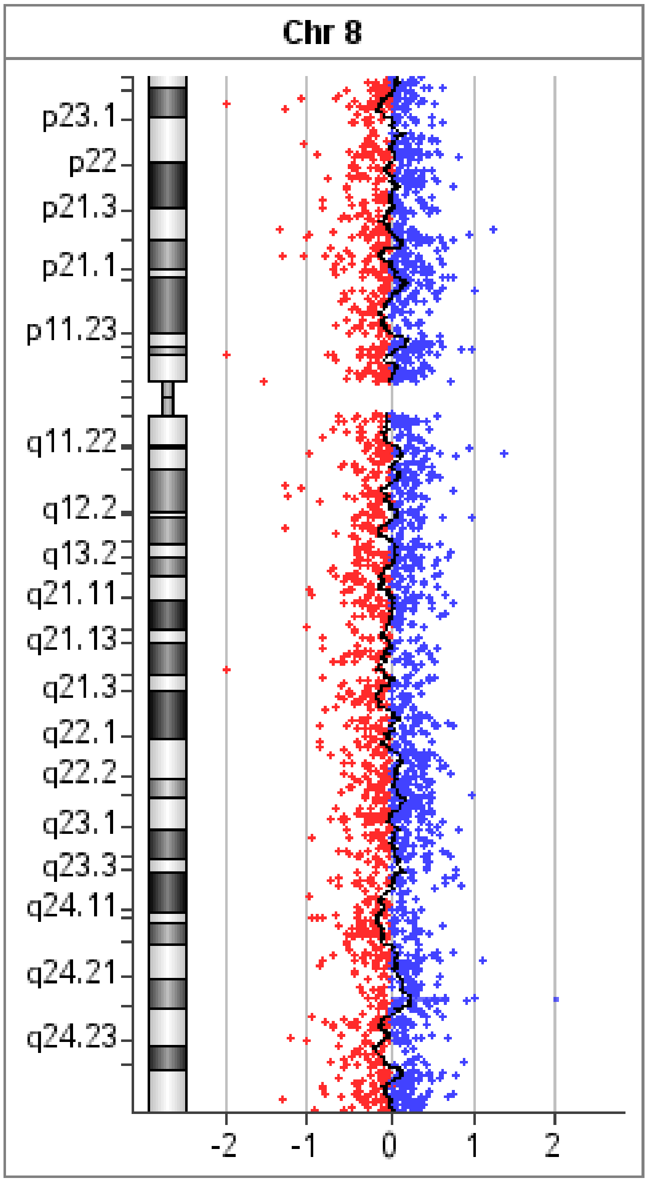

This is an intermediate report and not a final signed off report

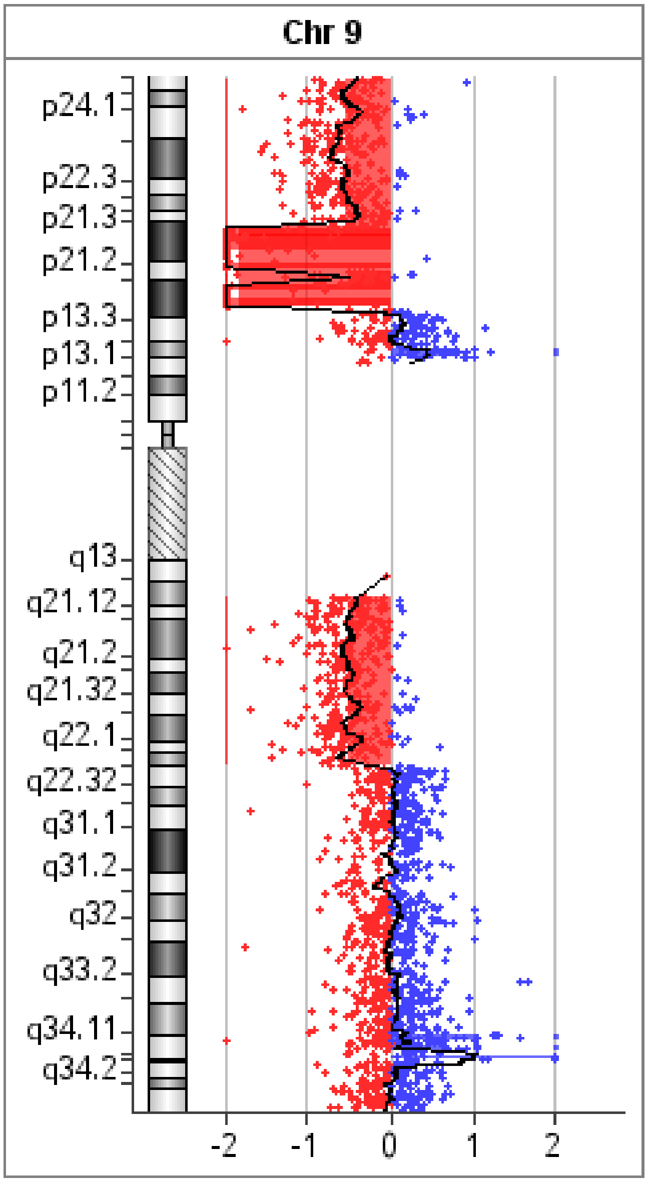

This is an intermediate report and not a final signed off report

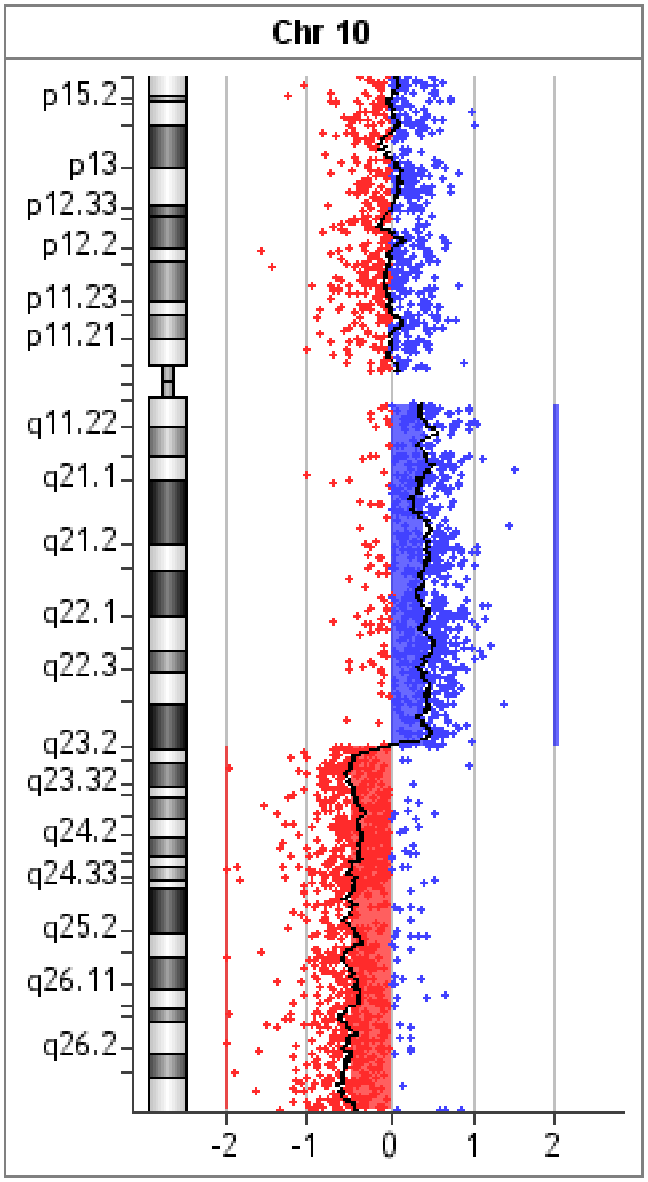

This is an intermediate report and not a final signed off report

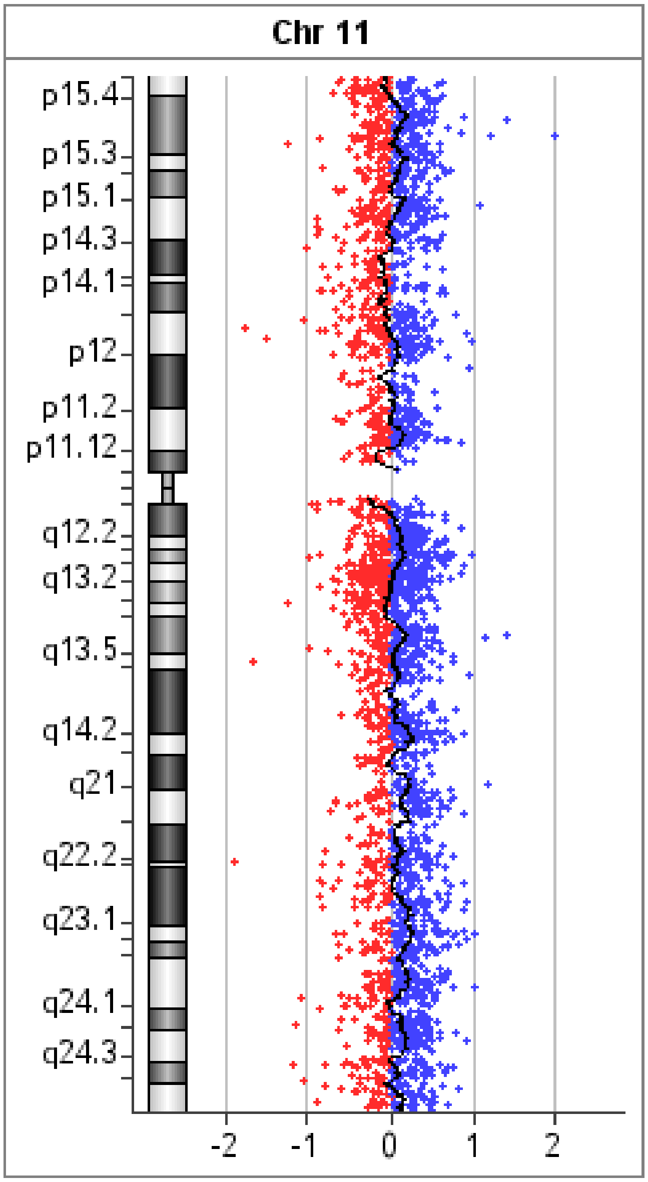

This is an intermediate report and not a final signed off report

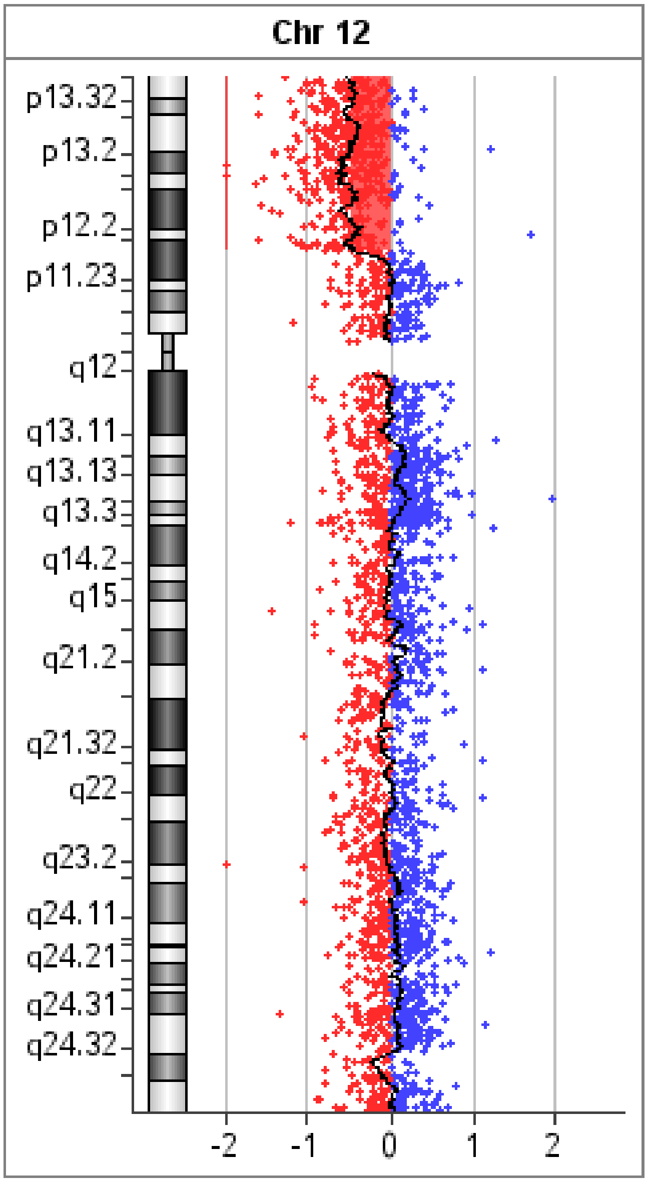

This is an intermediate report and not a final signed off report

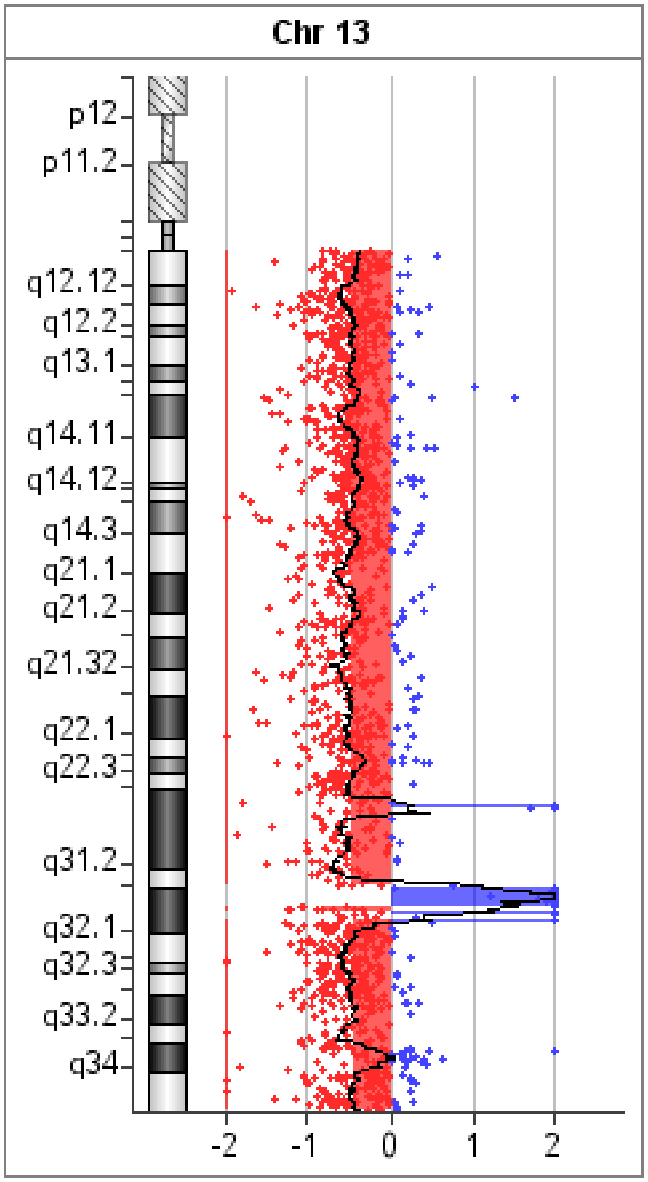

This is an intermediate report and not a final signed off report

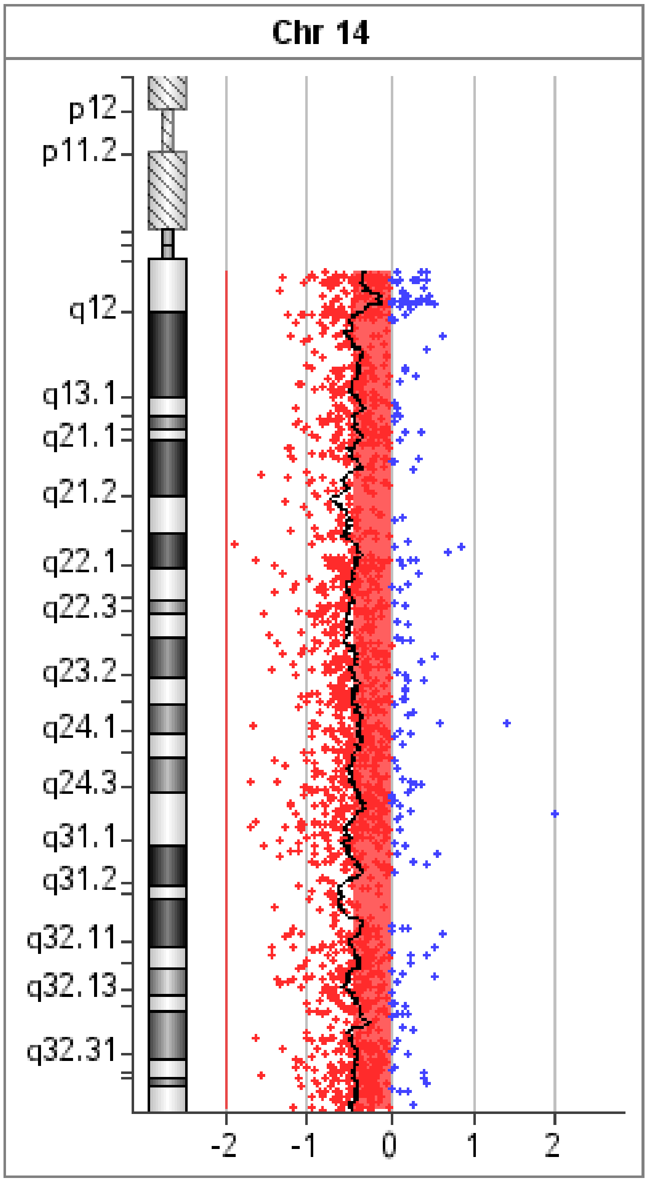

This is an intermediate report and not a final signed off report

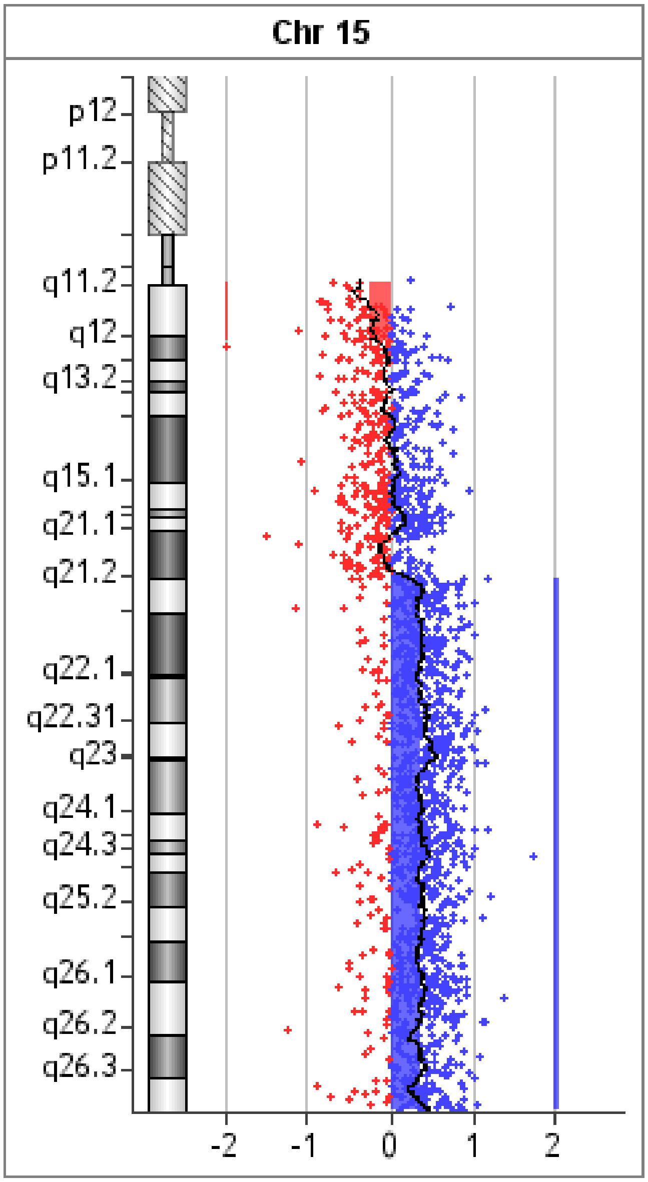

This is an intermediate report and not a final signed off report

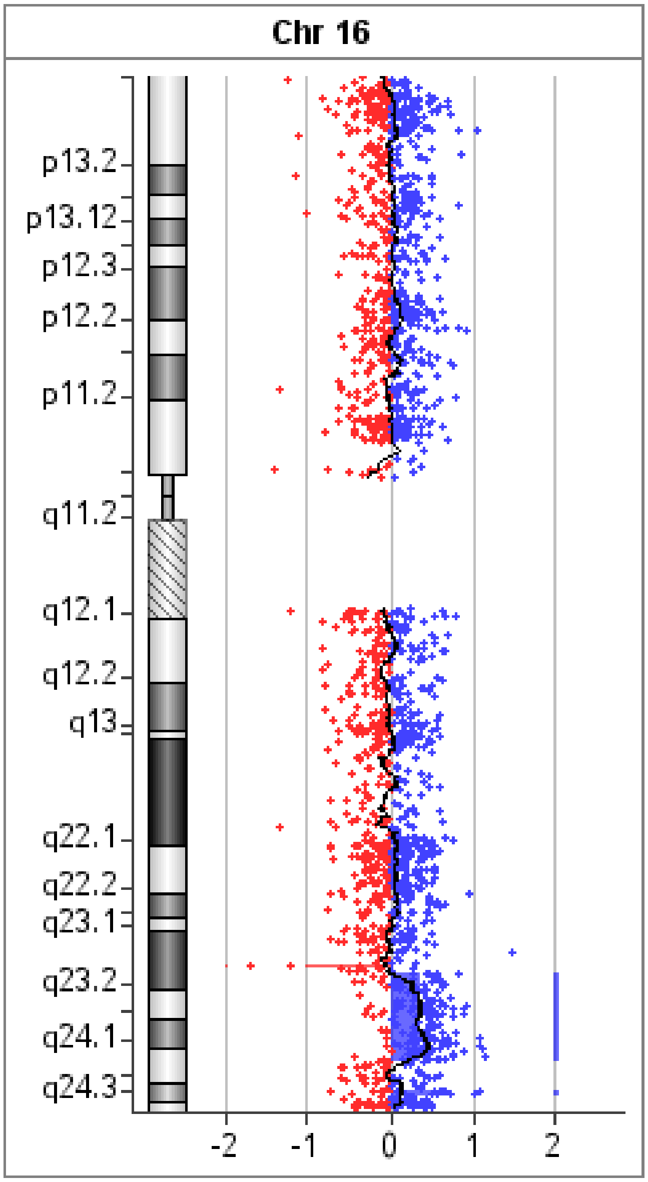

This is an intermediate report and not a final signed off report

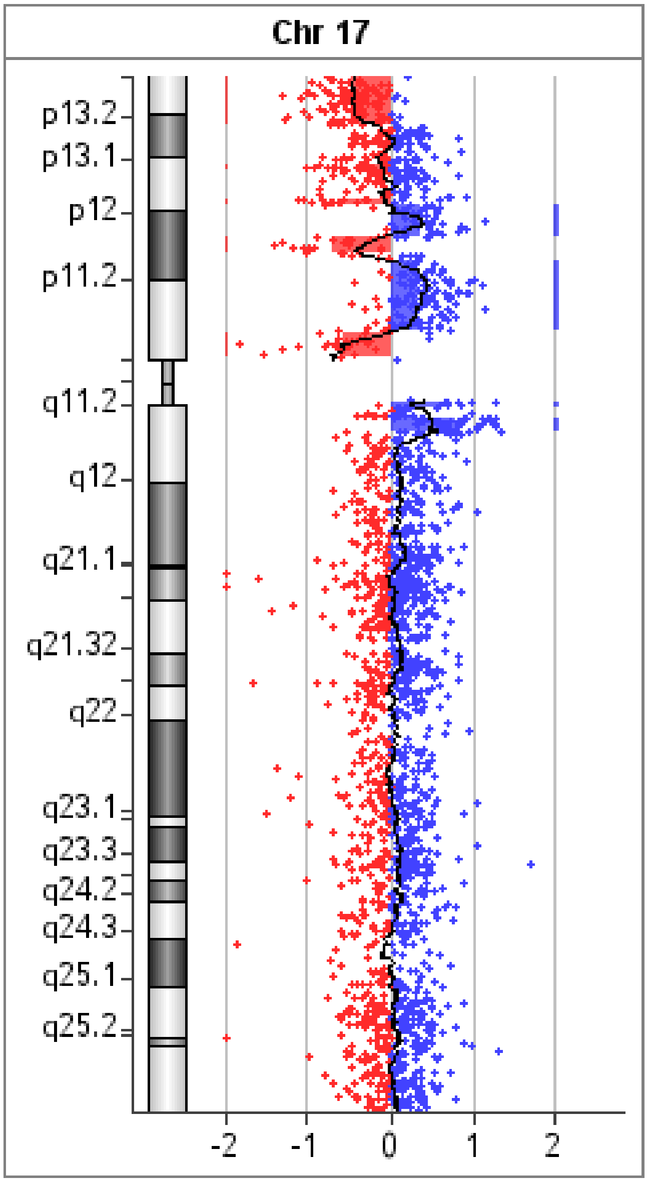

This is an intermediate report and not a final signed off report

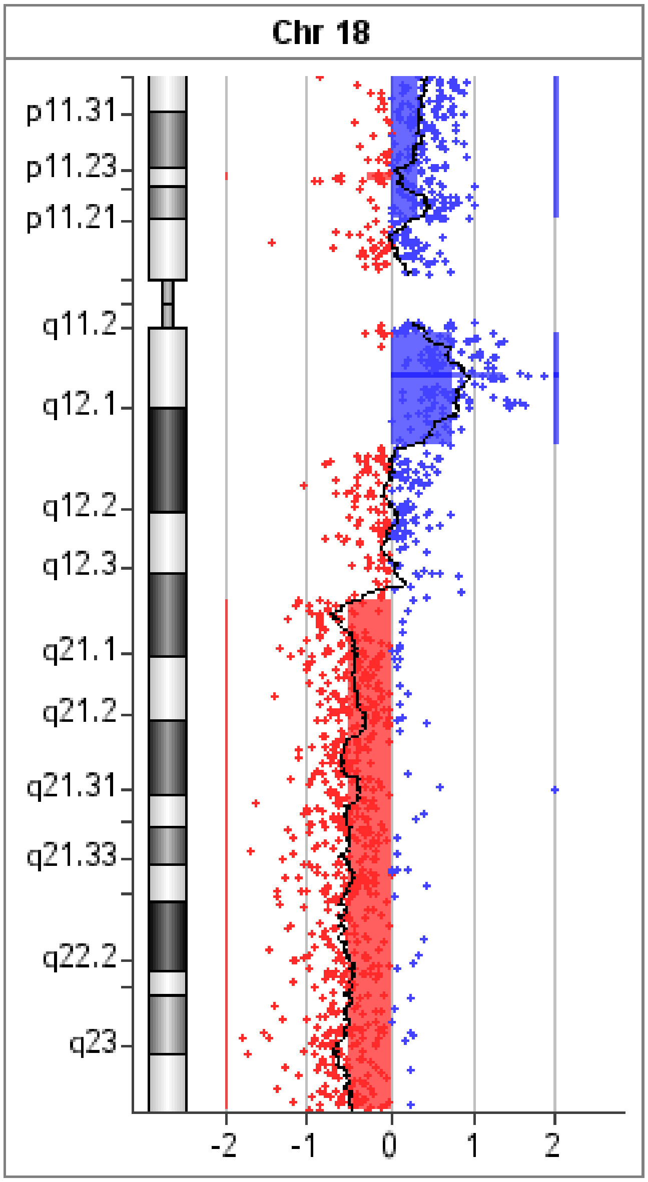

This is an intermediate report and not a final signed off report

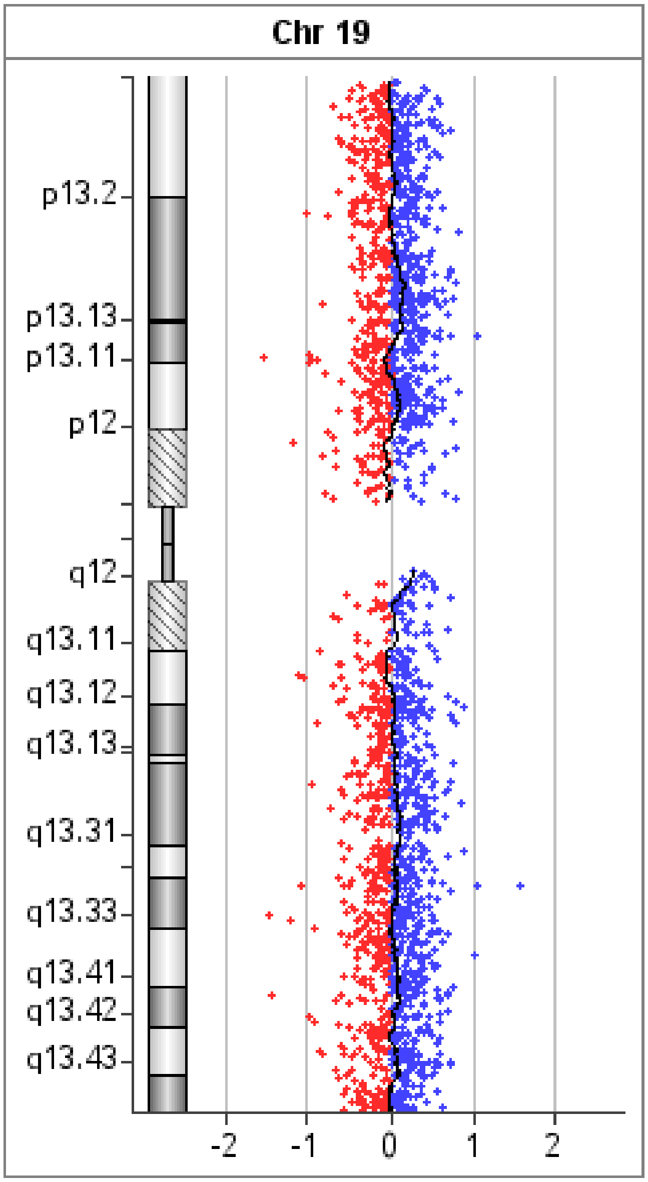

This is an intermediate report and not a final signed off report

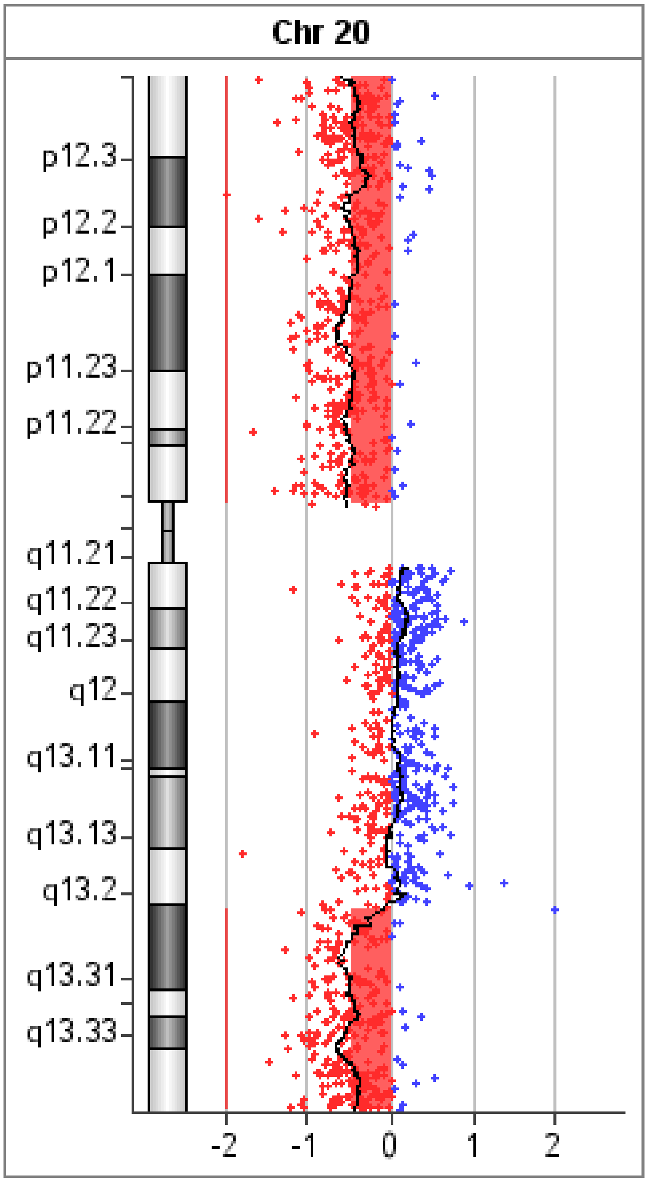

This is an intermediate report and not a final signed off report

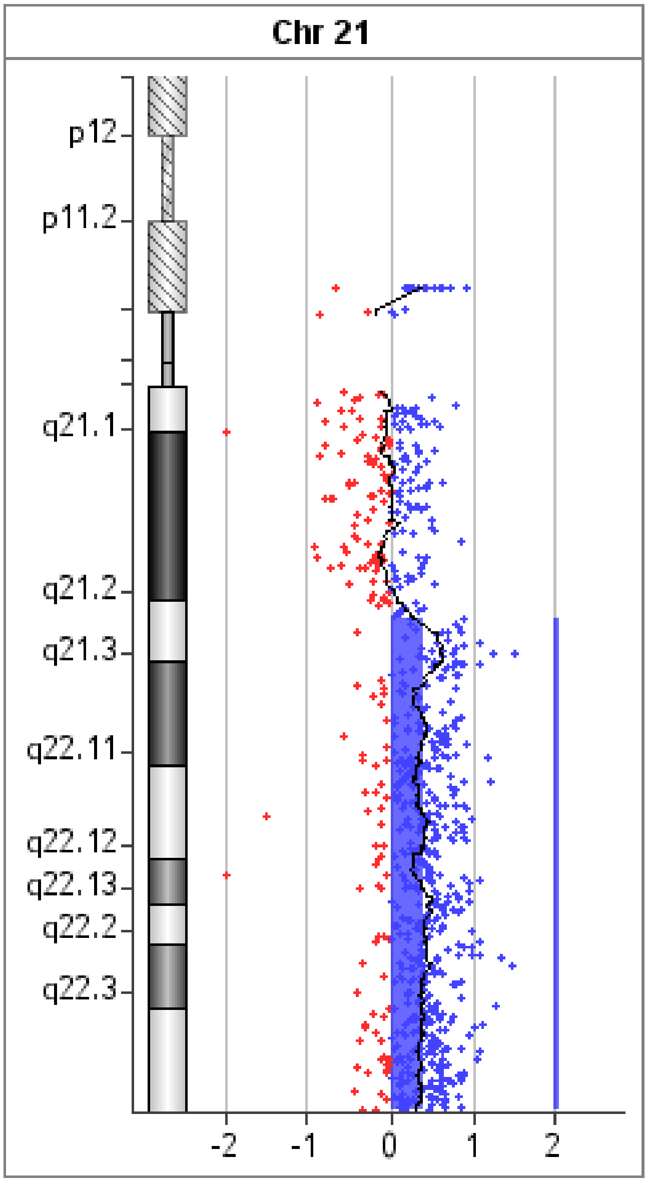

This is an intermediate report and not a final signed off report

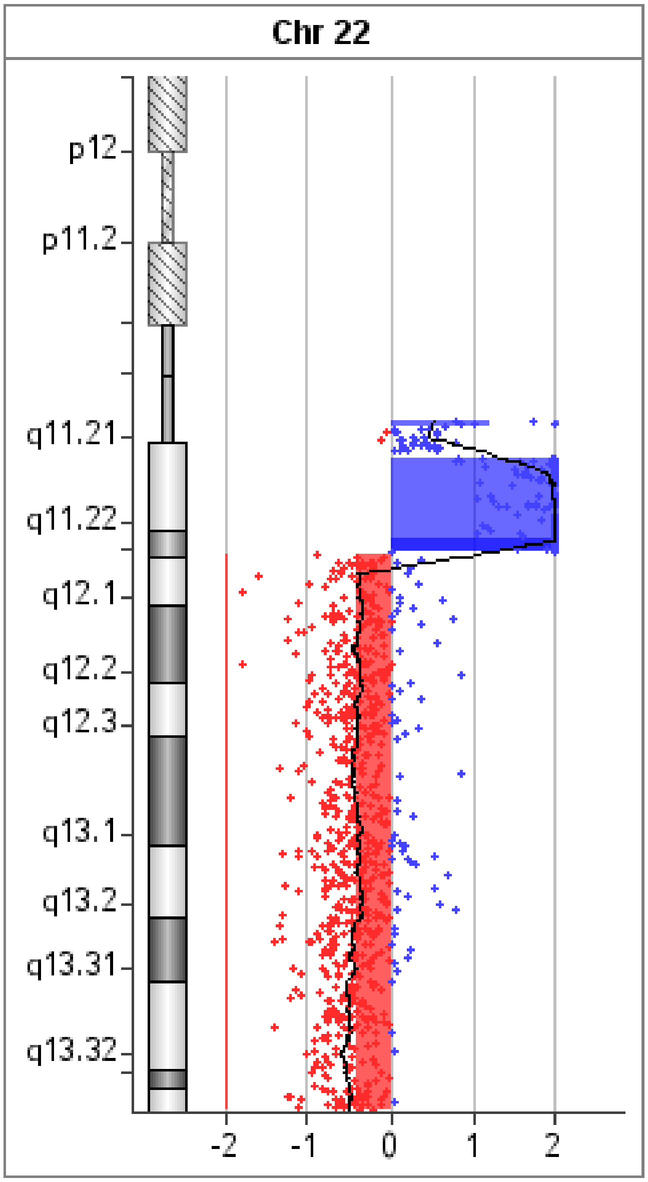

This is an intermediate report and not a final signed off report

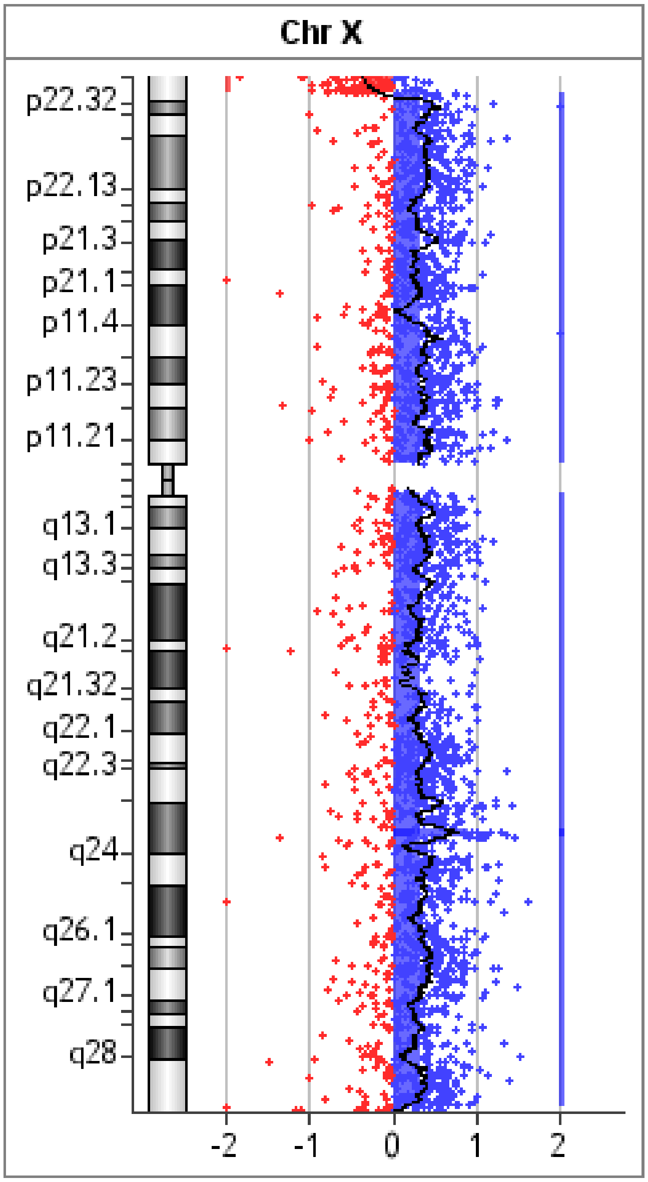

This is an intermediate report and not a final signed off report

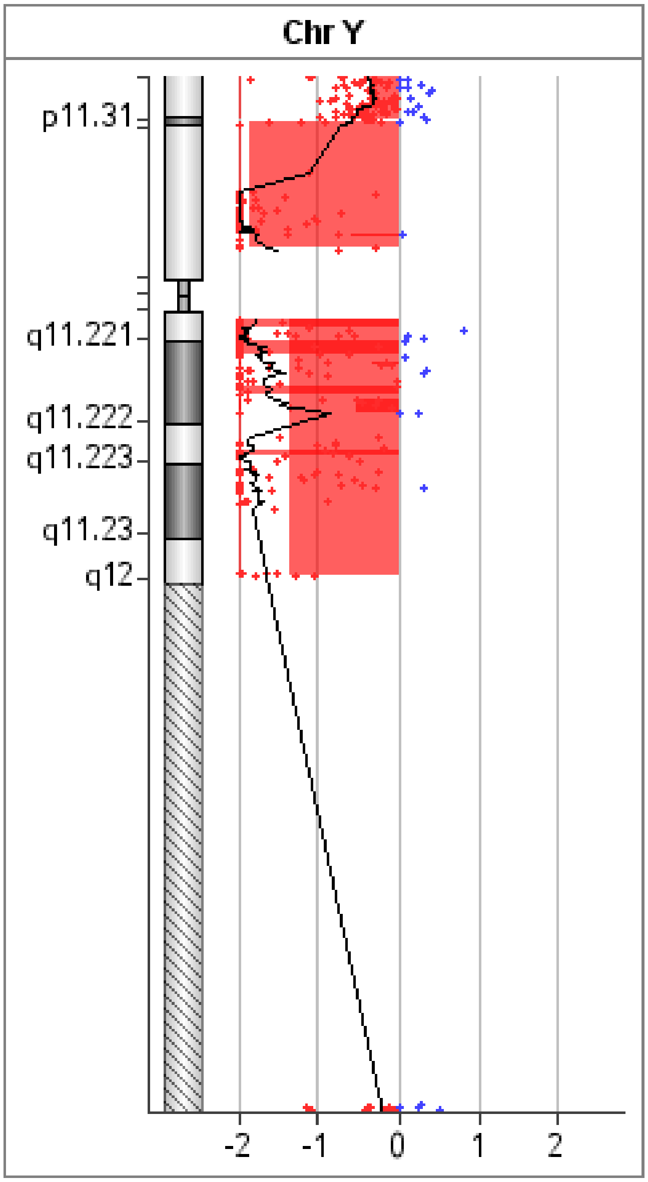

This is an intermediate report and not a final signed off report

Amp/Gain/Loss/Del Intervals Table

Chr	Start-Stop(bp)	Size(kb)	Cytoband	#Probes	Amp/Gain/Loss/Del	P-value	Annotations
chr1	852863-55252418	54,400	p36.33 - p32.3	1280	0.419838	4.90E-324	FLJ39609, SAMD11, NOC2L...
chr1	107600977-121281170	13,680	p13.3 - p11.2	312	0.379033	3.32E-105	PRMT6, NTNG1, VAV3...
chr1	162366109-249151859	86,786	q23.3 - q44	1734	0.381137	4.90E-324	SH2D1B, UHMK1, UAP1...
chr2	42444-91766271	91,724	p25.3 - p11.1	1610	-0.336081	4.90E-324	FAM110C, SH3YL1, ACP1...
chr2	206212-3338027	3,132	p25.3	53	-0.595295	4.32E-10	SH3YL1, ACP1, FAM150B...
chr2	18532630-23931404	5,399	p24.2 - p24.1	66	-0.628408	7.05E-15	RDH14, NT5C1B-RDH14, NT5C1B...
chr2	39990107-90069726	50,080	p22.1 - p11.2	888	-0.444143	1.08E-25	THUMPD2, SLC8A1, LOC388942...
chr2	149378946-188546067	39,167	q23.1 - q32.1	696	0.401962	1.36E-259	EPC2, KIF5C, LYPD6B...
chr2	149378946-150251368	872	q23.1 - q23.2	19	1.002411	7.95E-18	EPC2, KIF5C, LYPD6B...
chr2	189068304-225945697	36,877	q32.1 - q36.2	735	-0.436839	1.42E-319	GULP1, MIR561, DIRC1...
chr3	159711-48219787	48,060	p26.3 - p21.31	876	0.366380	1.30E-272	CHL1, CNTN6, CNTN4...
chr3	48456307-84777471	36,321	p21.31 - p12.1	704	-0.503400	4.90E-324	PLXNB1, CCDC51, CCDC72...
chr3	59764652-60055536	291	p14.2	10	-1.378287	9.21E-18	FHIT
chr3	78647198-80162698	1,516	p12.3 - p12.2	23	-0.906974	2.32E-10	ROBO1
chr3	93580736-197803820	104,223	q11.1 - q29	1899	-0.408153	4.90E-324	PROS1, ARL13B, STX19...
chr4	160517264-163435909	2,919	q32.1 - q32.2	30	-1.442988	5.26E-131	FSTL5
chr5	151737-46100367	45,949	p15.33 - p11	649	0.454856	6.07E-310	PLEKHG4B, LRRC14B, CCDC127...
chr5	364170-14011580	13,647	p15.33 - p15.2	183	0.659460	1.02E-19	AHRR, LOC100310782, C5orf55...
chr6	170426-51781749	51,611	p25.3 - p12.3	1145	0.445796	4.90E-324	DUSP22, IRF4, EXOC2...
chr6	31636310-31831356	195	p21.33	26	0.863127	2.97E-28	CSNK2B, LY6G5B, LY6G5C...

This is an intermediate report and not a final signed off report

Chr	Start-Stop(bp)	Size(kb)	Cytoband	#Probes	Amp/Gain/ Loss/Del	P-value	Annotations
chr6	36437799-51732931	15,295	p21.31 - p12.3	337	0.730621	2.70E-65	KCTD20, STK38, SRSF3...
chr6	87429974-92573234	5,143	q14.3 - q15	97	0.396478	4.48E-37	HTR1E, CGA, ZNF292...
chr6	108108995-110036365	1,927	q21	46	0.451328	1.72E-23	SCML4, SEC63, OSTM1...
chr6	135271127-170890108	35,619	q23.3 - q27	625	0.391941	7.26E-223	ALDH8A1, HBS1L, MIR3662...
chr7	62654304-159088636	96,434	q11.21 - q36.3	2053	0.548113	4.90E-324	LOC643955, LOC100287704, LOC100287834...
chr7	63108972-102114142	39,005	q11.21 - q22.1	747	0.330151	7.89E-84	ZNF727, ZNF735, ZNF679...
chr7	104343173-158909738	54,567	q22.2 - q36.3	1261	0.677689	6.74E-51	LHFPL3, LOC645591, LOC723809...
chr8	130425357-130788501	363	q24.21	9	0.641310	1.92E-10	GSDMC
chr9	373868-20718810	20,345	p24.3 - p21.3	326	-0.523864	6.18E-206	DOCK8, KANK1, DMRT1...
chr9	20737427-31510599	10,773	p21.3 - p21.1	153	-1.812013	4.90E-324	KIAA1797, PTPLAD2, IFNB1...
chr9	21002866-23724873	2,722	p21.3	53	-2.853239	1.12E-71	PTPLAD2, IFNB1, IFNW1...
chr9	21500692-21547395	47	p21.3	3	-4.911891	1.02E-10	LOC554202, MIR31
chr9	25583028-26465048	882	p21.3 - p21.2	8	-3.991042	1.70E-34	TUSC1
chr9	26649384-28428190	1,779	p21.2 - p21.1	35	-0.452039	3.20E-151	C9orf82, PLAA, IFT74...
chr9	28670476-29137041	467	p21.1	8	-3.841779	7.18E-31	LINGO2, MIR876, MIR873
chr9	30520898-31510599	990	p21.1	13	-2.738717	2.40E-16	
chr9	37288390-38403251	1,115	p13.2 - p13.1	27	0.579531	8.38E-23	ZCCHC7, GRHPR, ZBTB5...
chr9	71035346-94124443	23,089	q21.11 - q22.31	381	-0.489899	2.06E-211	PGM5, C9orf71, PIP5K1B...
chr9	130923464-131109445	186	q34.11	8	1.070250	3.25E-23	C9orf16, CIZ1, DNM1...
chr9	132436581-132612549	176	q34.11	8	0.698871	6.27E-11	PRRX2, PTGES, TOR1B...
chr9	133654917-134139531	485	q34.12 - q34.13	18	2.453455	1.32E-218	ABL1, QRFP, FIBCD1...
chr10	42976950-87825601	44,849	q11.21 - q23.1	746	0.399390	8.59E-276	LOC84856, ZNF37BP, ZNF33B...

This is an intermediate report and not a final signed off report

Chr	Start-Stop(bp)	Size(kb)	Cytoband	#Probes	Amp/Gain/Loss/Del	P-value	Annotations
chr10	87892105-135234843	47,343	q23.1 - q26.3	972	-0.468740	4.90E-324	GRID1, MIR346, WAPAL...
chr12	230421-22695049	22,465	p13.33 - p12.1	520	-0.475779	1.62E-267	IQSEC3, LOC574538, SLC6A12...
chr13	19296544-89960396	70,664	q11 - q31.2	1206	-0.468881	4.90E-324	LOC284232, LOC348021, PHF2P1...
chr13	81137986-81454116	316	q31.1	5	2.446169	1.76E-97	
chr13	90457836-92416896	1,959	q31.3	34	2.336517	4.90E-324	MIR622, LOC144776, MIR17HG...
chr13	92494334-92892587	398	q31.3	11	-0.791542	2.24E-16	GPC5
chr13	92973314-93309763	336	q31.3	9	2.576738	5.31E-138	GPC5
chr13	93879390-93989566	110	q31.3	4	2.601232	9.06E-61	GPC6
chr13	94035921-114946264	20,910	q31.3 - q34	440	-0.434579	3.32E-191	GPC6, DCT, TGDS...
chr14	20472548-107258824	86,786	q11.2 - q32.33	1726	-0.432687	4.90E-324	OR4K14, OR4K13, OR4L1...
chr15	20481702-26191904	5,710	q11.1 - q12	68	-0.259966	1.66E-12	HERC2P3, GOLGA6L6, GOLGA8C...
chr15	49834437-102465355	52,631	q21.2 - q26.3	1148	0.366844	4.90E-324	C15orf33, DTWD1, ATP8B4...
chr16	77549380-77789804	240	q23.1	4	-1.019041	1.09E-10	NUDT7
chr16	78283614-86053065	7,769	q23.1 - q24.1	173	0.349224	8.17E-51	WWOX, MAF, DYNLRB2...
chr16	88653937-88810033	156	q24.2 - q24.3	11	0.606487	2.95E-11	ZC3H18, IL17C, CYBA...
chr17	148092-3962522	3,814	p13.3 - p13.2	120	-0.457988	4.82E-60	RPH3AL, C17orf97, FAM101B...
chr17	6913780-7367196	453	p13.1	31	-0.353722	7.32E-11	ALOX12, RNASEK, RNASEK-C17ORF49...
chr17	9739759-10049224	309	p13.1	16	-0.584165	1.41E-14	GLP2R, RCVRN, GAS7
chr17	10127678-12608962	2,481	p13.1 - p12	46	0.357461	1.49E-15	MYH13, MYH8, MYH4...
chr17	12759033-14013054	1,254	p12	22	-0.691451	8.56E-26	ARHGAP44, ELAC2, HS3ST3A1...
chr17	14569547-19959014	5,389	p12 - p11.2	131	0.342193	2.19E-37	CDRT7, PMP22, TEKT3...
chr17	20141913-22078577	1,937	p11.2	28	-0.560215	1.70E-21	SPECC1, CCDC144C, LGALS9B...

This is an intermediate report and not a final signed off report

Chr	Start-Stop(bp)	Size(kb)	Cytoband	#Probes	Amp/Gain/Loss/Del	P-value	Annotations
chr17	25590352-25954807	364	q11.1 - q11.2	10	0.687910	7.19E-13	WSB1, LOC440419, KSR1
chr17	26883246-27926708	1,043	q11.2	45	0.767973	1.90E-63	PIGS, ALDOC, SPAG5...
chr18	142096-10855438	10,713	p11.32 - p11.22	200	0.323401	1.88E-50	USP14, THOC1, COLEC12...
chr18	7438812-8015690	577	p11.23	13	-0.269840	1.68E-12	PTPRM
chr18	19506073-27890123	8,384	q11.2 - q12.1	129	0.746234	1.74E-167	GATA6, CTAGE1, RBBP8...
chr18	22400125-22934634	535	q11.2	10	1.368583	7.54E-11	ZNF521
chr18	39537606-77982126	38,445	q12.3 - q23	639	-0.504090	4.90E-324	PIK3C3, RIT2, SYT4...
chr20	121521-26128919	26,007	p13 - p11.1	487	-0.475429	6.28E-254	DEFB126, DEFB127, DEFB128...
chr20	50788962-62872839	12,084	q13.2 - q13.33	264	-0.477664	1.12E-140	ZFP64, TSHZ2, ZNF217...
chr21	25203765-48018909	22,815	q21.2 - q22.3	545	0.384308	5.96E-187	NCRNA00158, MIR155HG, MIR155...
chr22	17096855-17407203	310	q11.1	6	1.202907	4.29E-22	psiTPTE22, XKR3, HSFY1P1
chr22	18953012-23627391	4,674	q11.21 - q11.23	124	2.065511	4.90E-324	DGCR5, DGCR9, DGCR10...
chr22	22998284-23627391	629	q11.22 - q11.23	18	2.755399	1.96E-17	MIR650, IGLL5, RTDR1...
chr22	23739437-51178264	27,439	q11.23 - q13.33	738	-0.420082	1.42E-301	ZDHHC8P1, IGLL1, C22orf43...
chrX	61091-2608349	2,547	p22.33	91	-0.344257	1.16E-26	PLCXD1, GTPBP6, NCRNA00107...
chrX	2709027-58051765	55,343	p22.33 - p11.21	989	0.338837	2.26E-256	XG, GYG2, ARSD...
chrX	62645308-154664396	92,019	q11.1 - q28	1573	0.339561	4.90E-324	LOC92249, ARHGEF9, MIR1468...
chrX	112926208-114305500	1,379	q23	26	0.903381	8.78E-21	HTR2C, SNORA35, MIR764...
chrY	11091-2558349	2,547	p11.32 - p11.31	91	-0.344257	1.16E-26	PLCXD1, GTPBP6, NCRNA00107...
chrY	2694871-9901314	7,206	p11.31 - p11.2	58	-1.860709	2.39E-273	RPS4Y1, ZFY, TGIF2LY...
chrY	9042367-9148336	106	p11.2	3	-0.585518	4.08E-11	
chrY	14061053-28767604	14,707	q11.21 - q11.23	144	-1.343495	4.90E-324	GYG2P1, TTTY15, USP9Y...
chrY	14061053-14463950	403	q11.21	7	-2.786670	3.11E-18	
chrY	15206937-15977687	771	q11.221	15	-2.141166	8.54E-15	UTY, TMSB4Y

This is an intermediate report and not a final signed off report

Chr	Start-Stop(bp)	Size(kb)	Cytoband	#Probes	Amp/Gain/Loss/Del	P-value	Annotations
chrY	16393891-16640171	246	q11.221	4	-0.328297	1.95E-11	NLGN4Y
chrY	17890214-18255512	365	q11.221	5	-2.590785	3.21E-10	
chrY	18574255-19291733	717	q11.221	9	-0.497090	8.28E-17	
chrY	21491899-21843986	352	q11.222	6	-2.877162	5.38E-14	BCORP1, CYorf15A, CYorf15B

Amp=Amplification Del=Deletion

Total Amp/Gain/Loss/Del Intervals: 92

This is an intermediate report and not a final signed off report

Analysis Settings

Design	:	021924_20101001
Genome	:	hg19
Threshold	:	6.0
GC Correction	:	ON
Centralization (legacy)	:	OFF
SNP Copy Number	:	OFF
Combine Replicates (Intra Array)	:	ON
Metric Set Filter	:	NONE
Feature Level Filter	:	gIsSaturated = true OR rIsSaturated = true OR gIsFeatNonUnifOL = true OR rIsFeatNonUnifOL = true OR LogRatio = 0; Include matching values=false
LOH Filter	:	NONE
Show Flat Intervals	:	false

Sample Name	:	252192435816_2_3
Aberration Algorithm	:	ADM-2
Fuzzy Zero	:	OFF
Window Size	:	2Kb
Diploid Peak Centralization	:	ON
LOH	:	OFF
Array Level Filter	:	NONE
Aberration Filter	:	Minimum Number of Probes for Amplification >= 3 AND Nesting Level <= 100 AND Minimum Avg. Absolute Log Ratio for Amplification >= 0.25 AND Minimum Size (Kb) of Region for Amplification >= 0.0 AND Minimum Size (Kb) of Region for Deletion >= 0.0 AND Minimum Number of Probes for Deletion >= 3 AND Minimum Avg. Absolute Log Ratio for Deletion >= 0.25 AND Minimum Number of Probes for Gain >= 3 AND Minimum Number of Probes for Loss >= 3 AND Minimum Avg. Absolute Log Ratio for Gain >= 0.25 AND Minimum Avg. Absolute Log Ratio for Loss >= 0.25 AND Minimum Size (Kb) of Region for Gain >= 0.0 AND Minimum Size (Kb) of Region for Loss >= 0.0
Design Level Filter	:	Homology = 0 OR IsPseudoautosomal = 1
Genomic Boundary	:	OFF
Template Name	:	Default Cyto Report Template - CGH

This is an intermediate report and not a final signed off report

Notes

Sample Notes

No notes available.

Amp/Gain/Loss/Del Interval Notes

No notes available.

This is an intermediate report and not a final signed off report
