# Supplementary material for: DNA Fingerprint Analysis of Raman Spectra Captures Global Genomic Alterations in Imatinib-Resistant Chronic Myeloid Leukemia: A Potential Single Assay for Screening Imatinib Resistance
Source: Cells. 2021 Sep 22;10(10):2506. doi: 10.3390/cells10102506 (PMC8533852; doi:10.3390/cells10102506)
Supplement: Supplementary file 1 [file cells-10-02506-s001.zip › Report S2-K562-S aCGH profile.pdf]

Sample Information

Green Sample	:	Research K562-S
Array ID	:	252192435816_2_2
Global Display Name	:	252192435816_2_2
Polarity	:	-1
Red Sample	:	Reference DNA
DerivativeOfLogRatioSD	:	0.386728
Intermediate Report by	:	DATASYSTEM\admin

Genome View (Amp/Del)

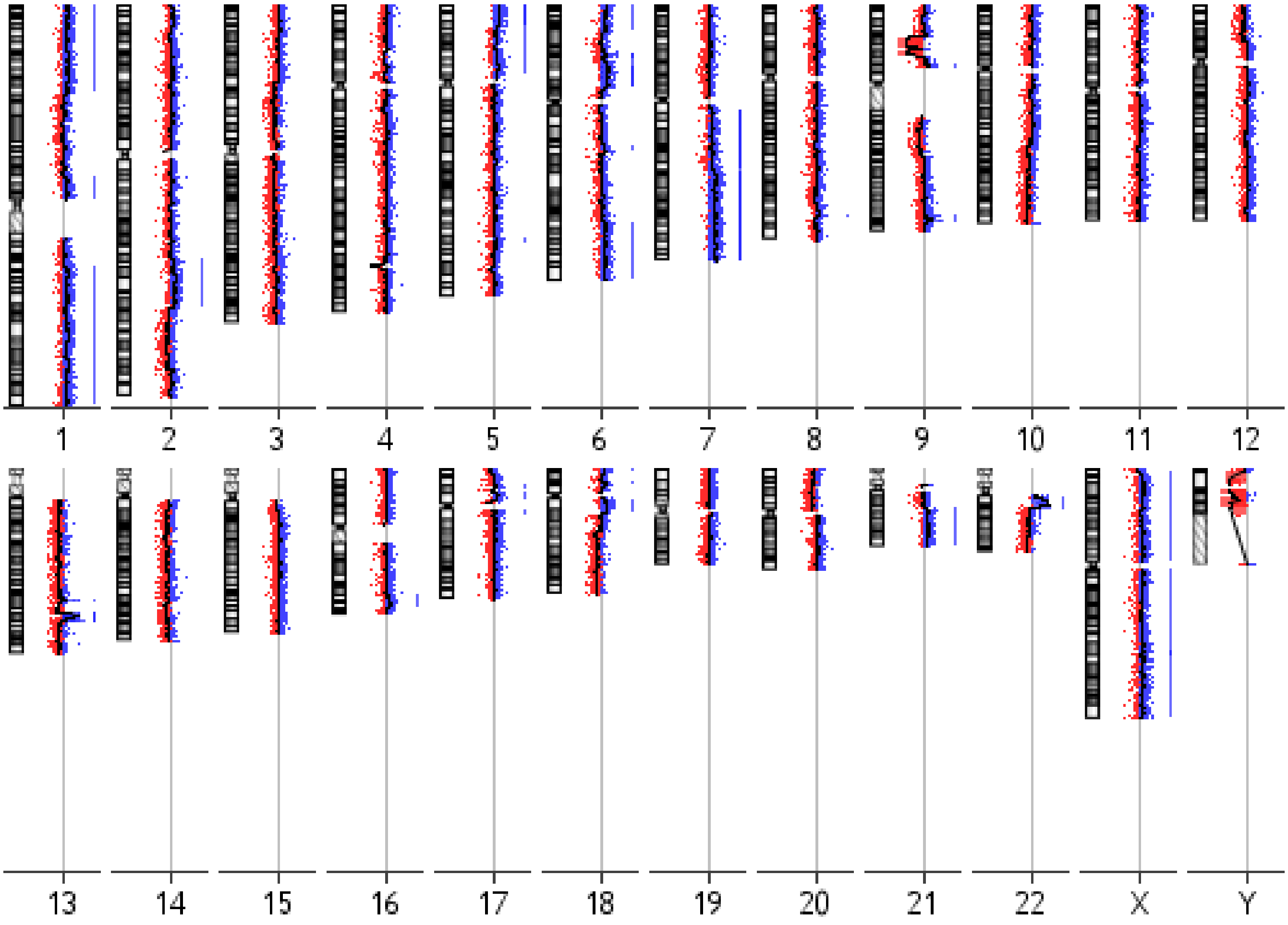

This is an intermediate report and not a final signed off report

Chromosome Views (Amp/Del)

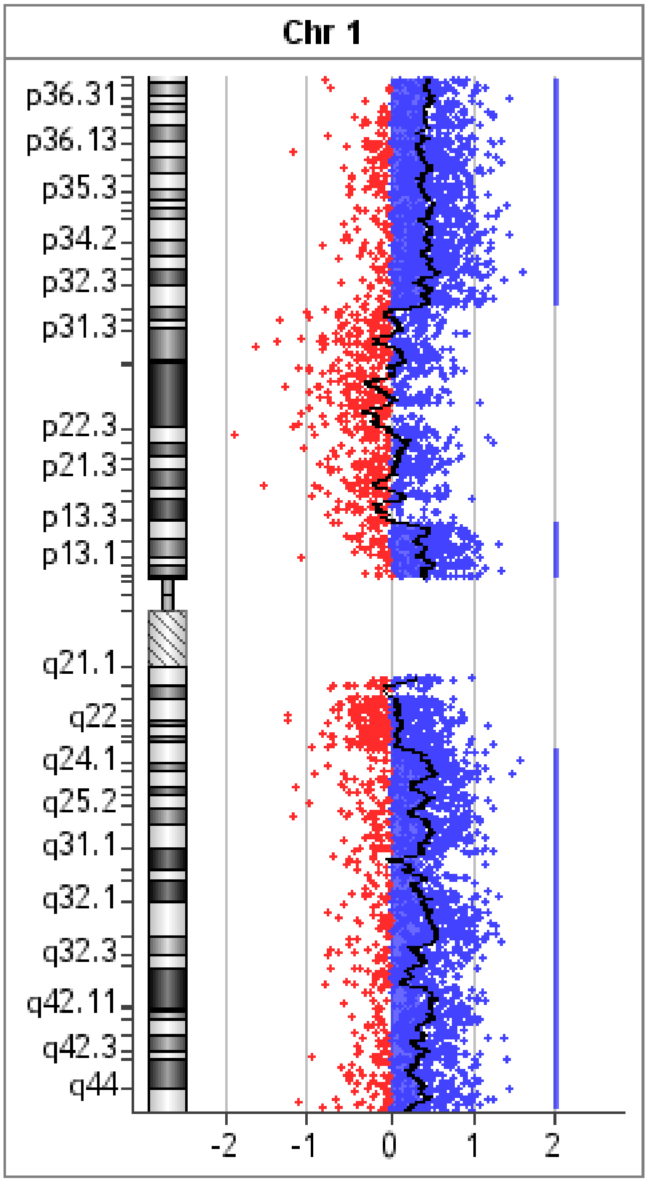

This is an intermediate report and not a final signed off report

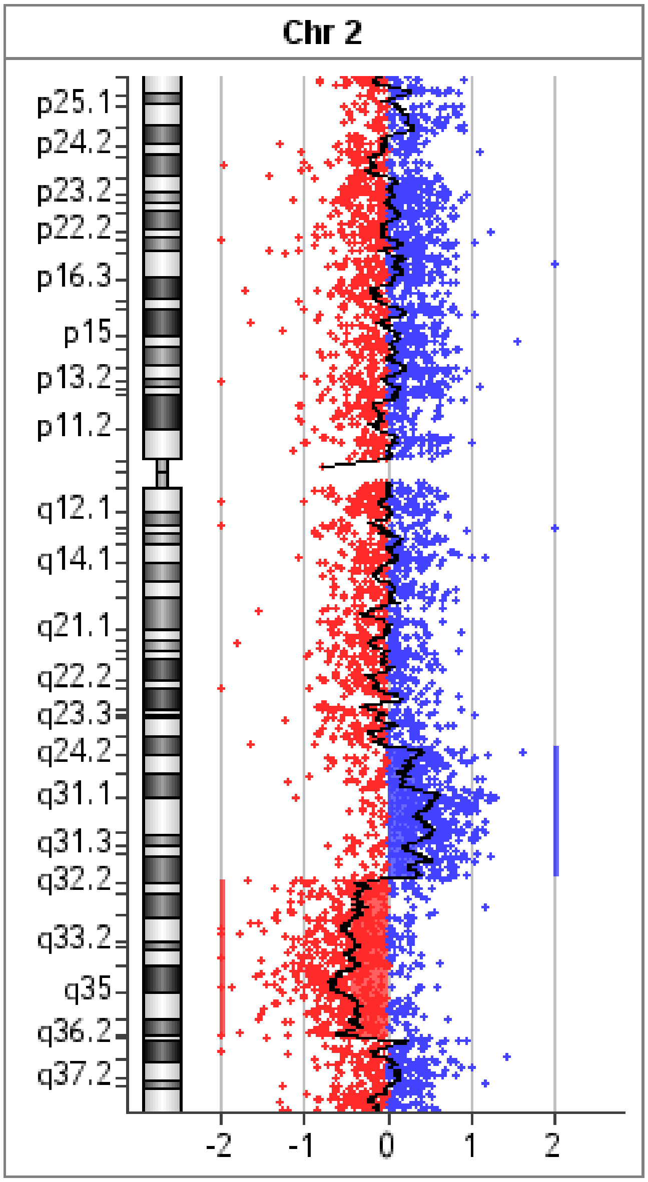

This is an intermediate report and not a final signed off report

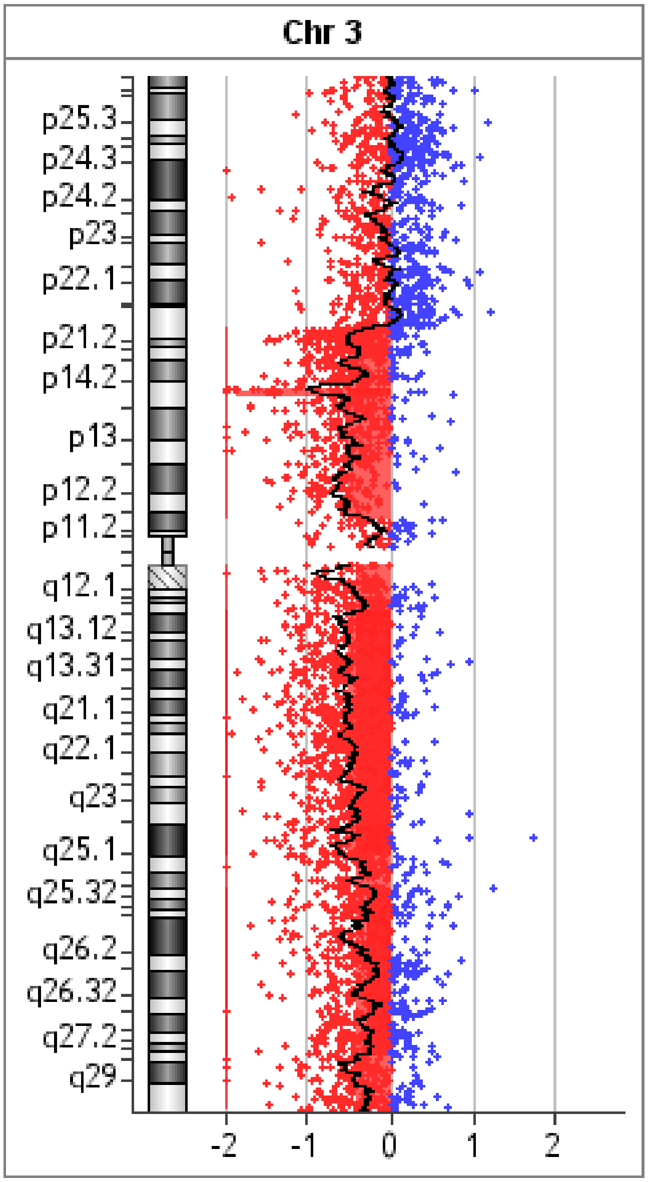

This is an intermediate report and not a final signed off report

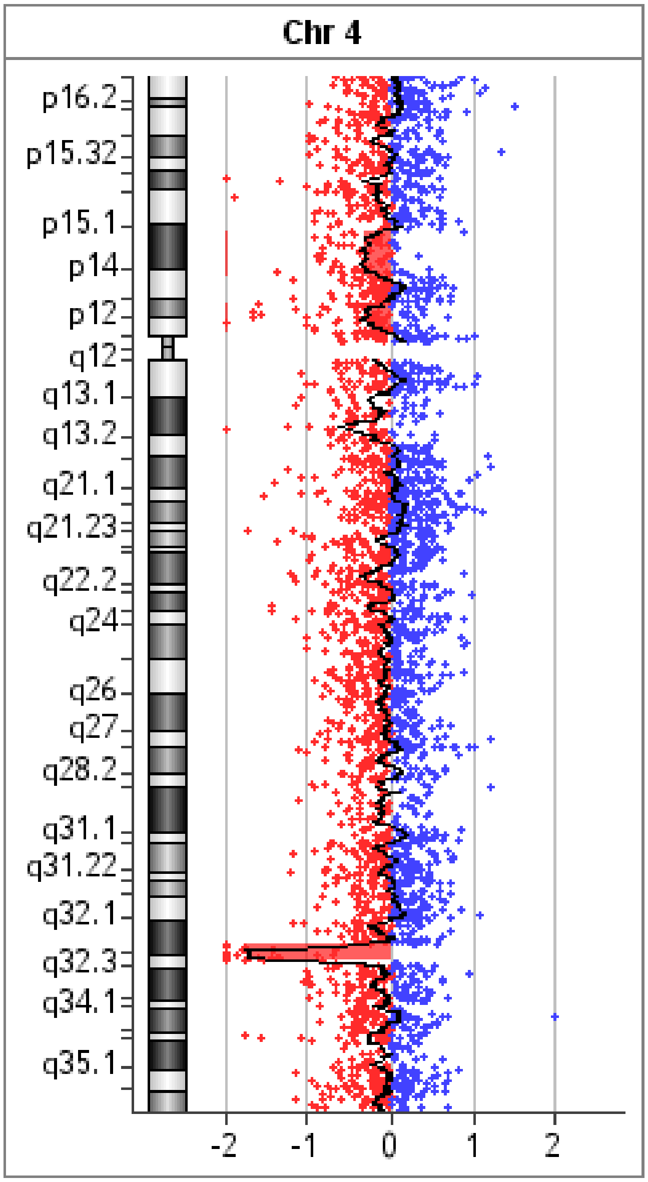

This is an intermediate report and not a final signed off report

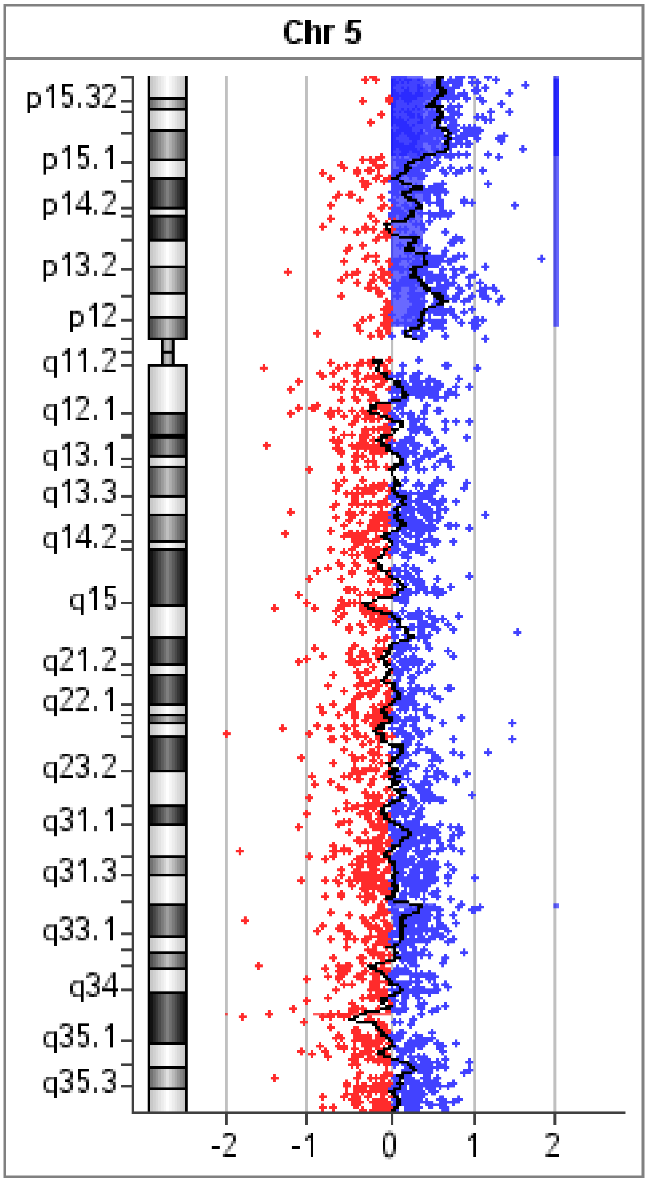

This is an intermediate report and not a final signed off report

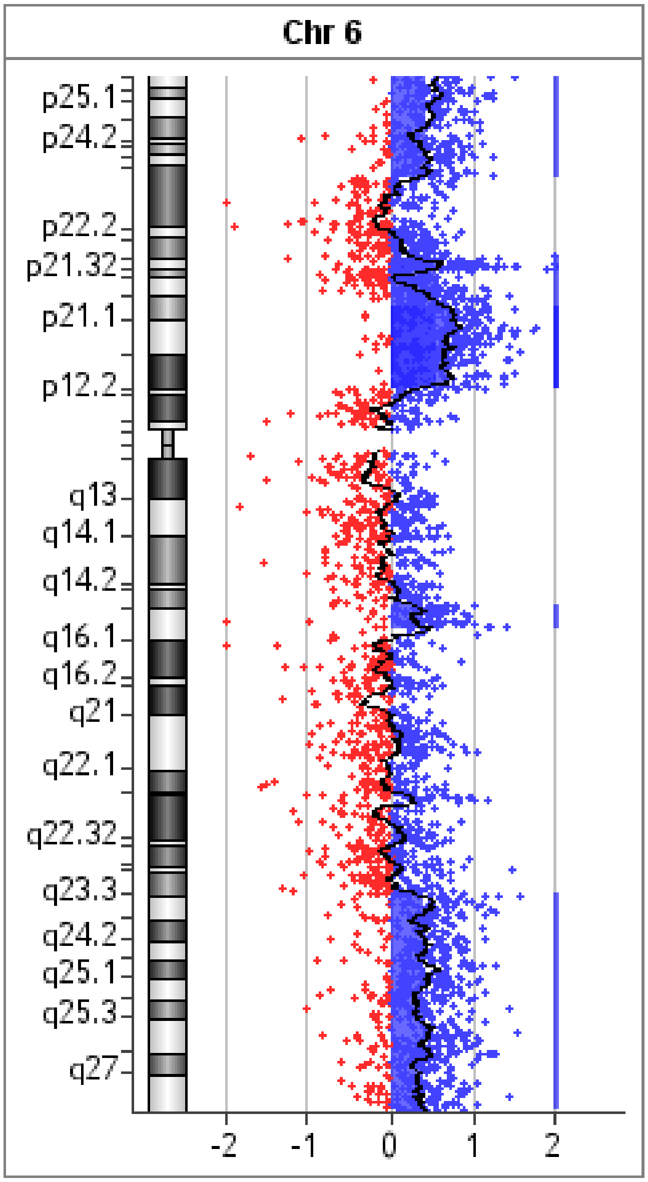

This is an intermediate report and not a final signed off report

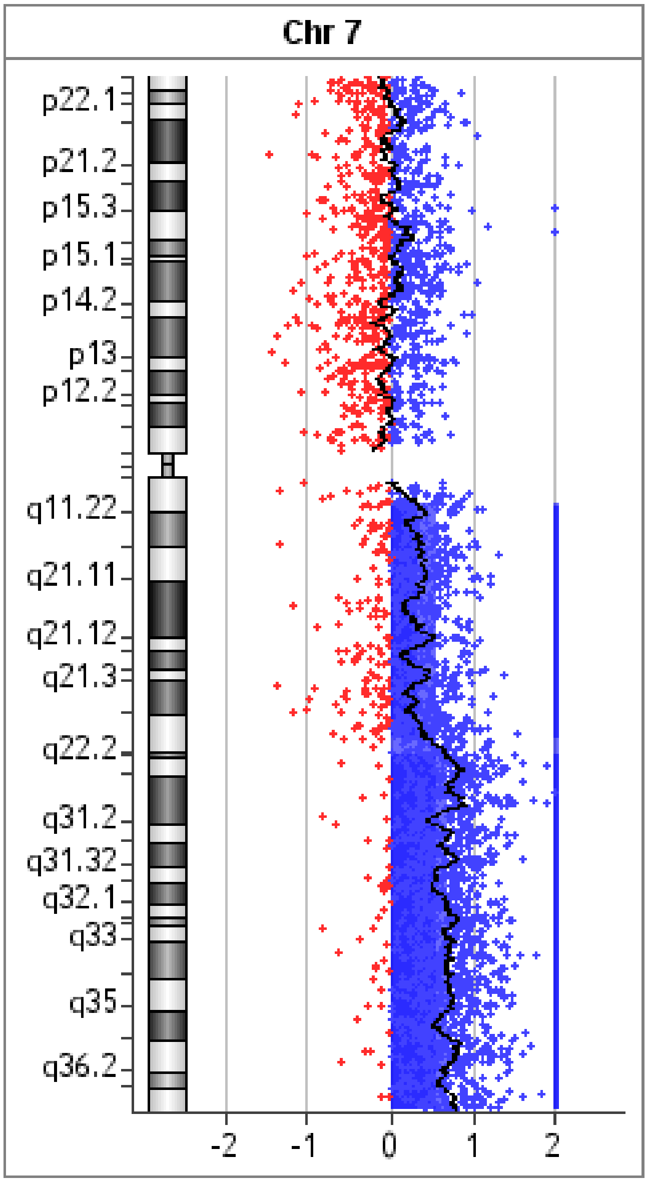

This is an intermediate report and not a final signed off report

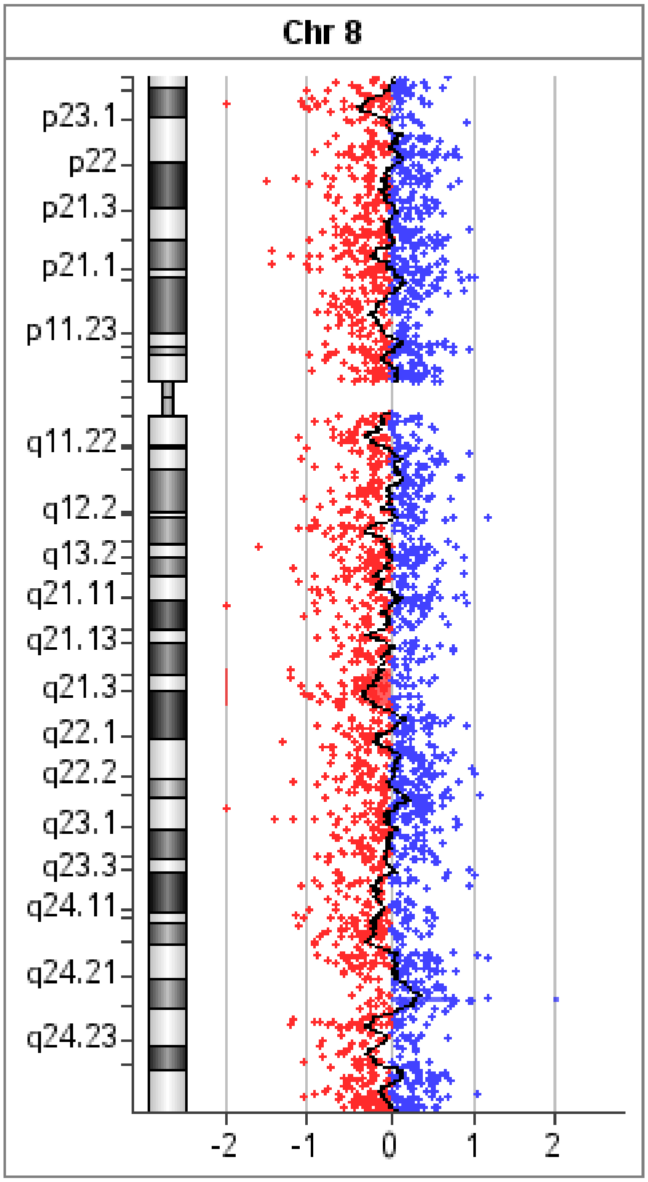

This is an intermediate report and not a final signed off report

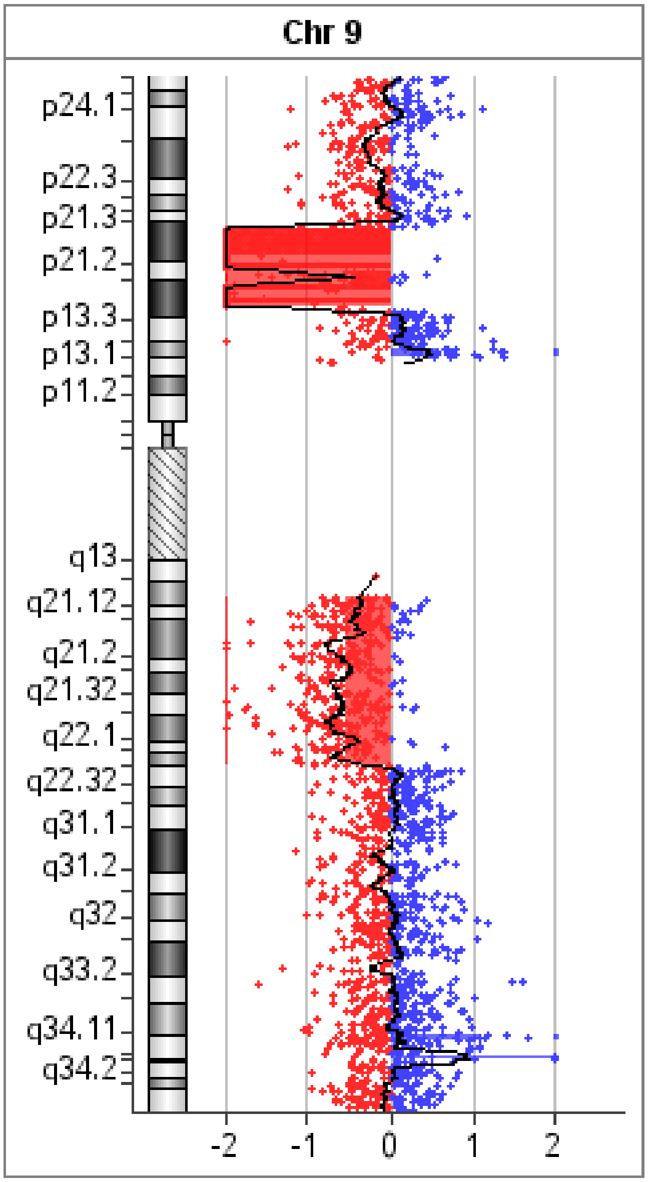

This is an intermediate report and not a final signed off report

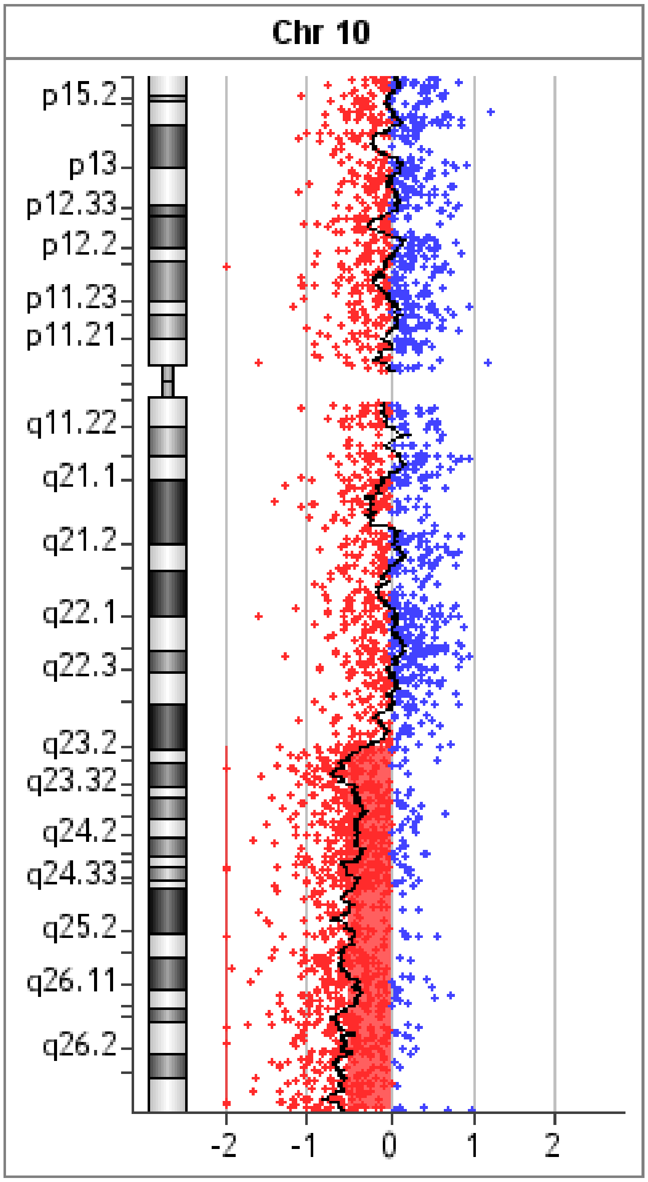

This is an intermediate report and not a final signed off report

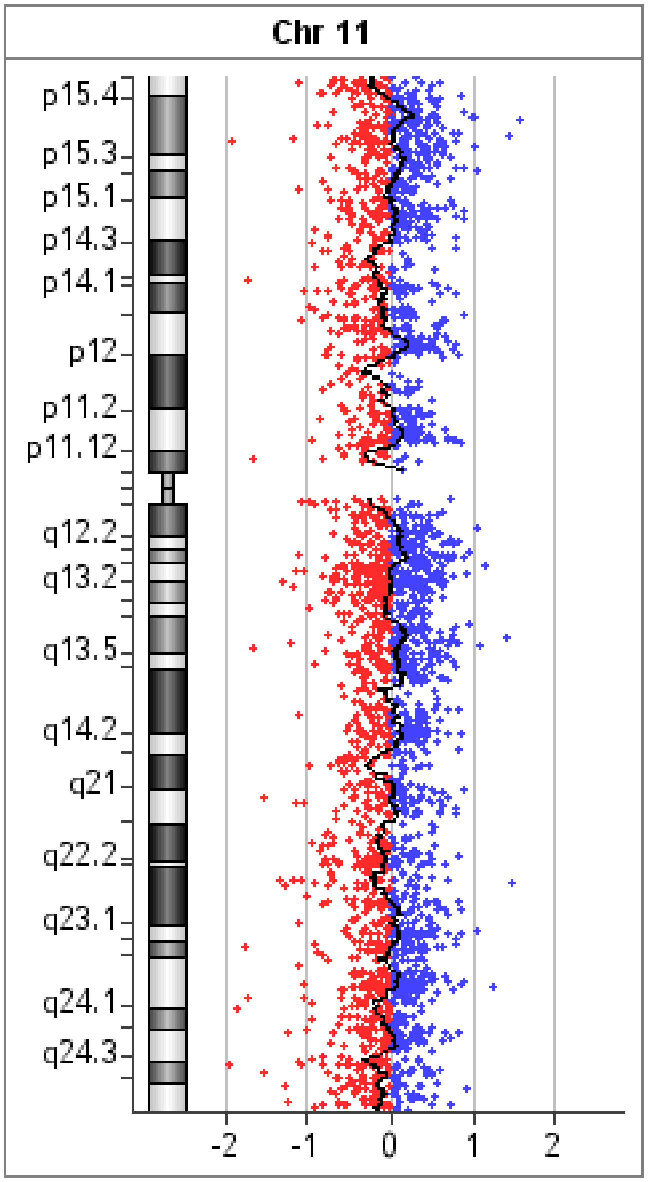

This is an intermediate report and not a final signed off report

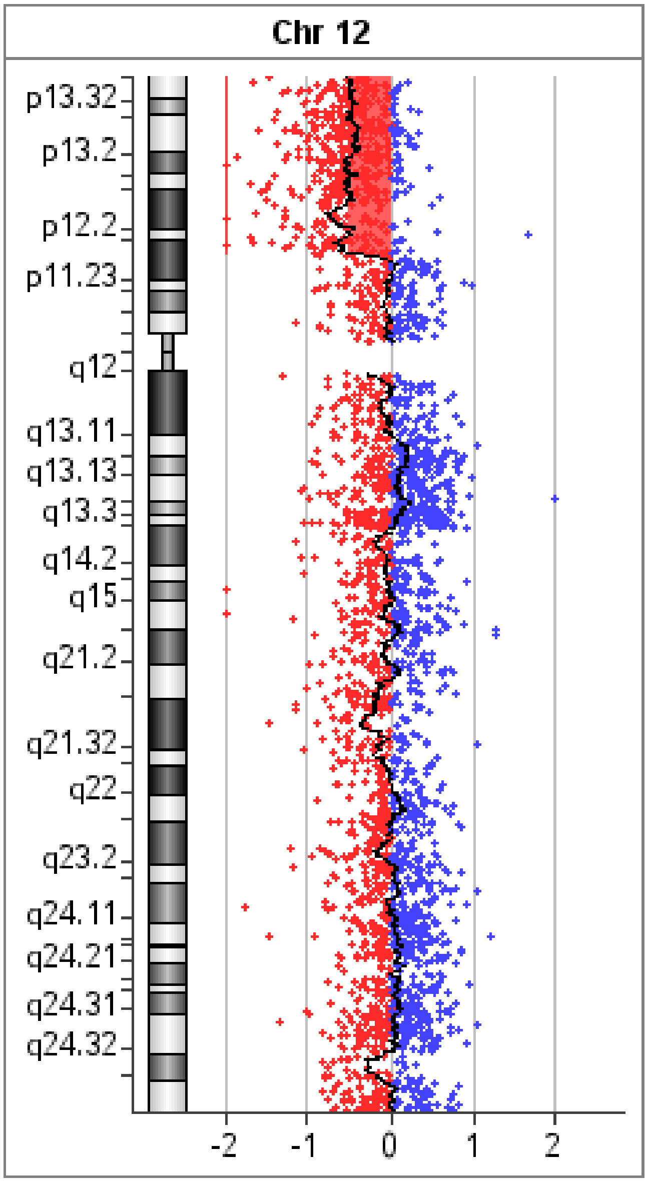

This is an intermediate report and not a final signed off report

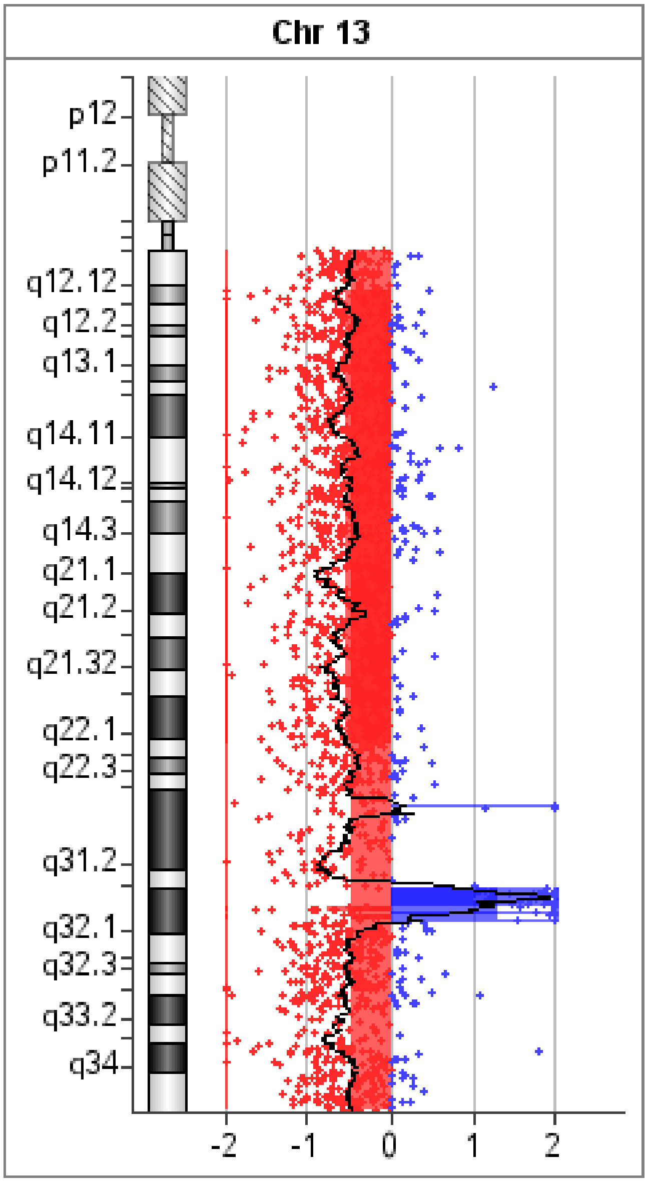

This is an intermediate report and not a final signed off report

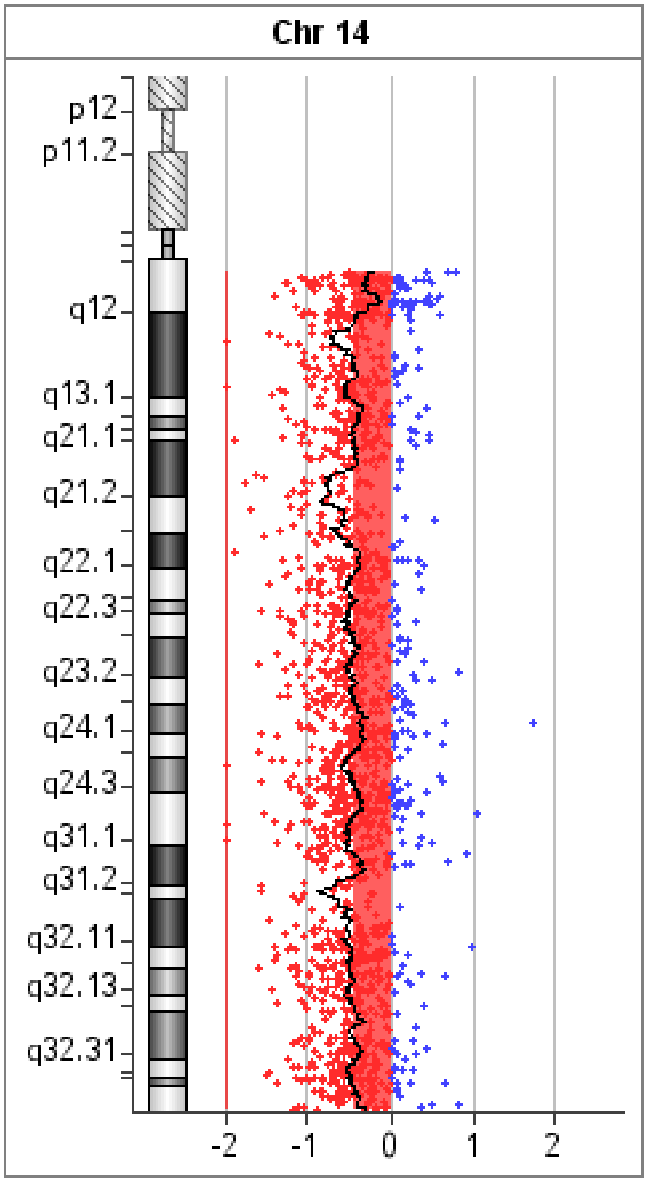

This is an intermediate report and not a final signed off report

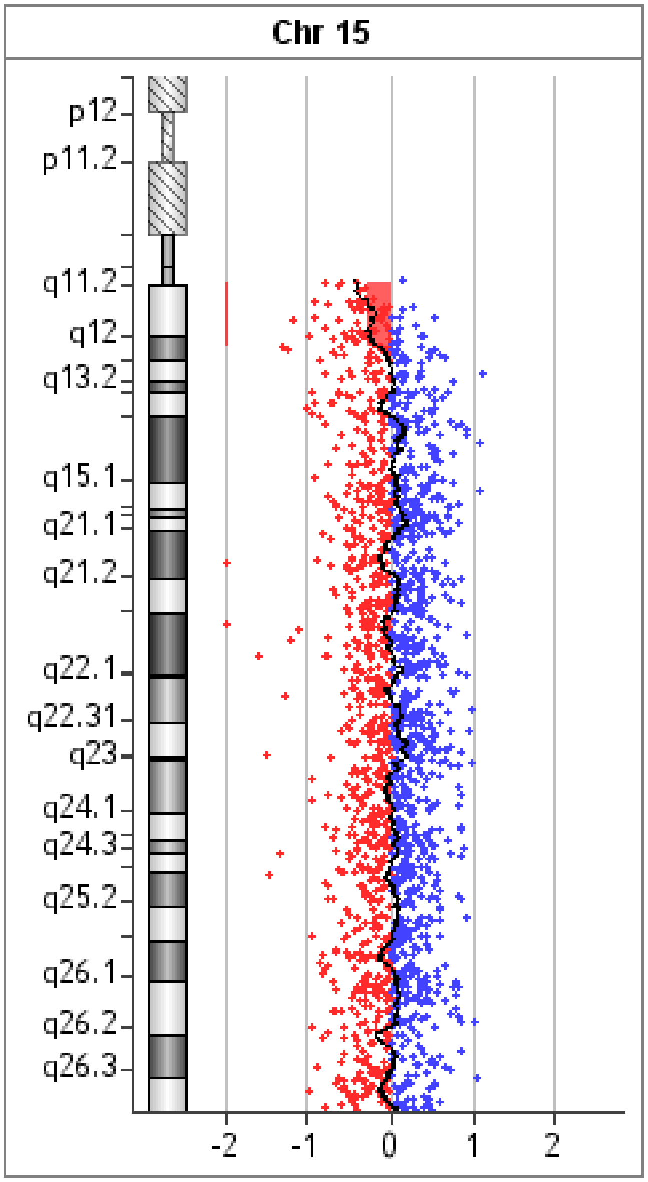

This is an intermediate report and not a final signed off report

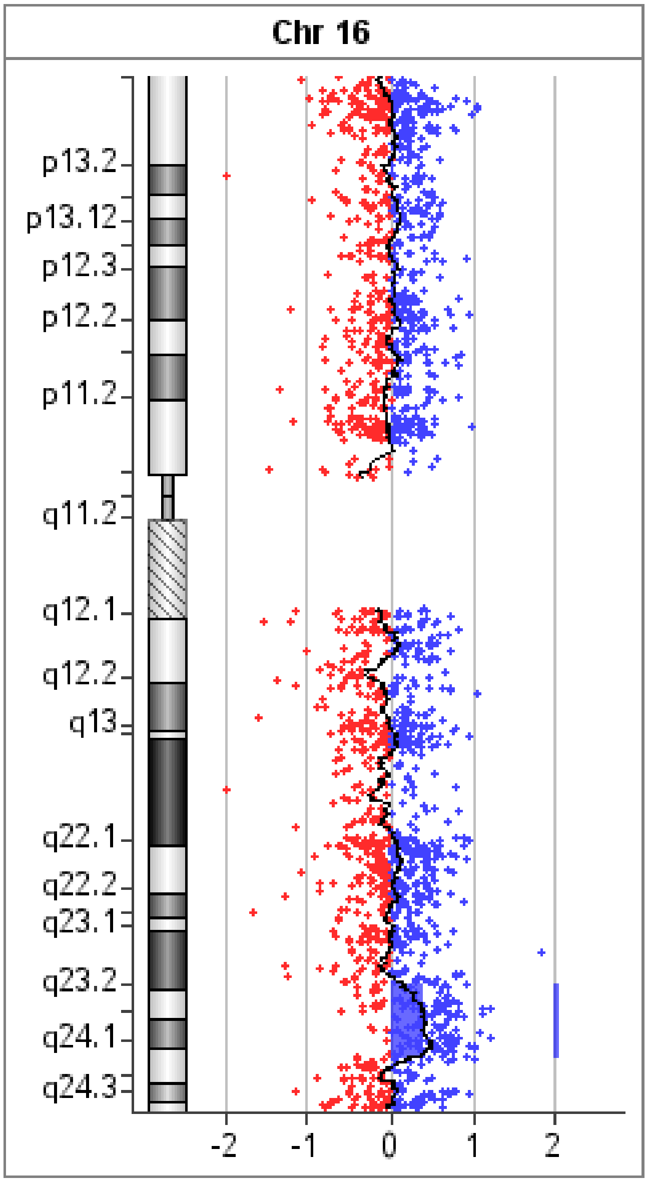

This is an intermediate report and not a final signed off report

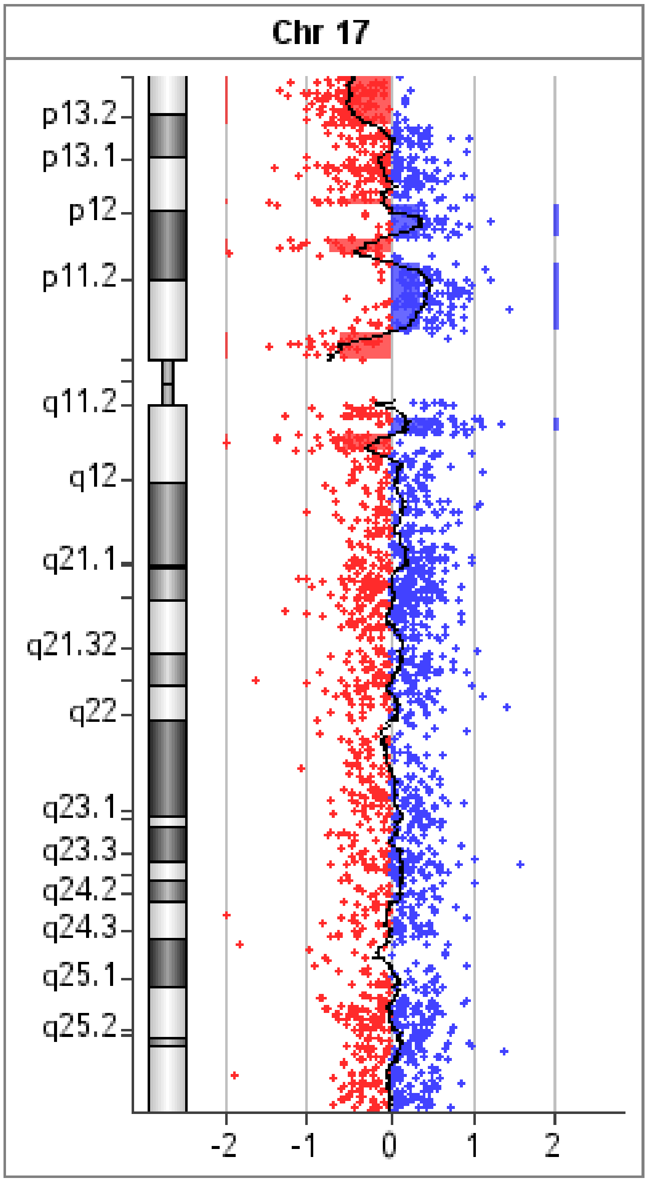

This is an intermediate report and not a final signed off report

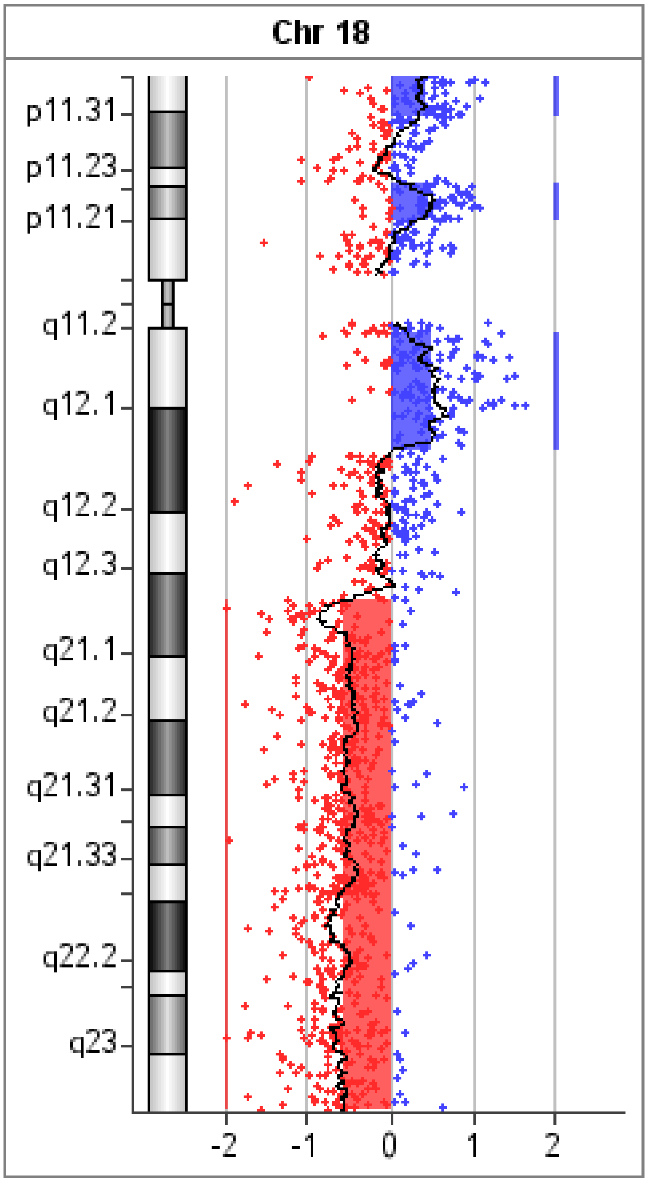

This is an intermediate report and not a final signed off report

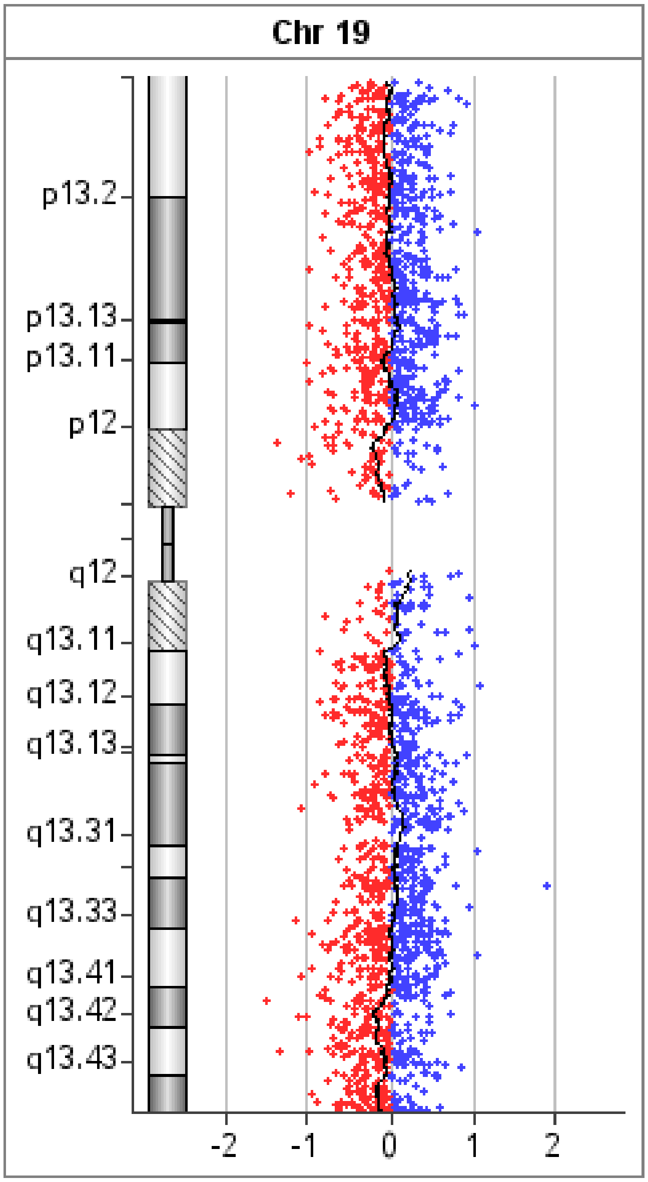

This is an intermediate report and not a final signed off report

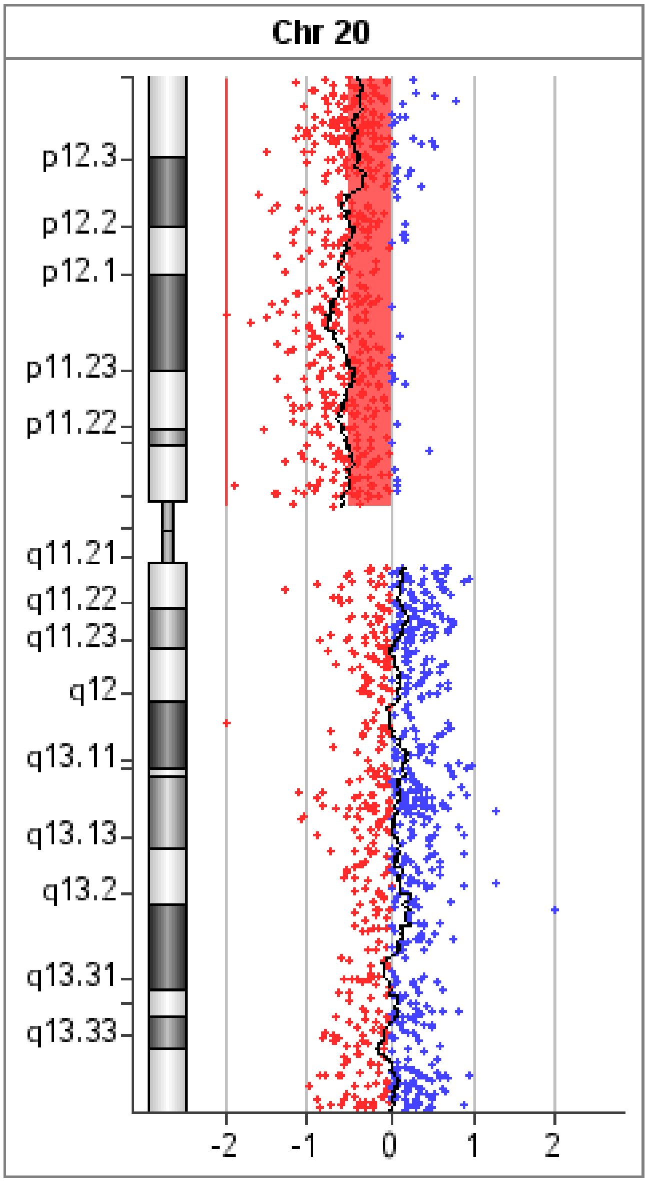

This is an intermediate report and not a final signed off report

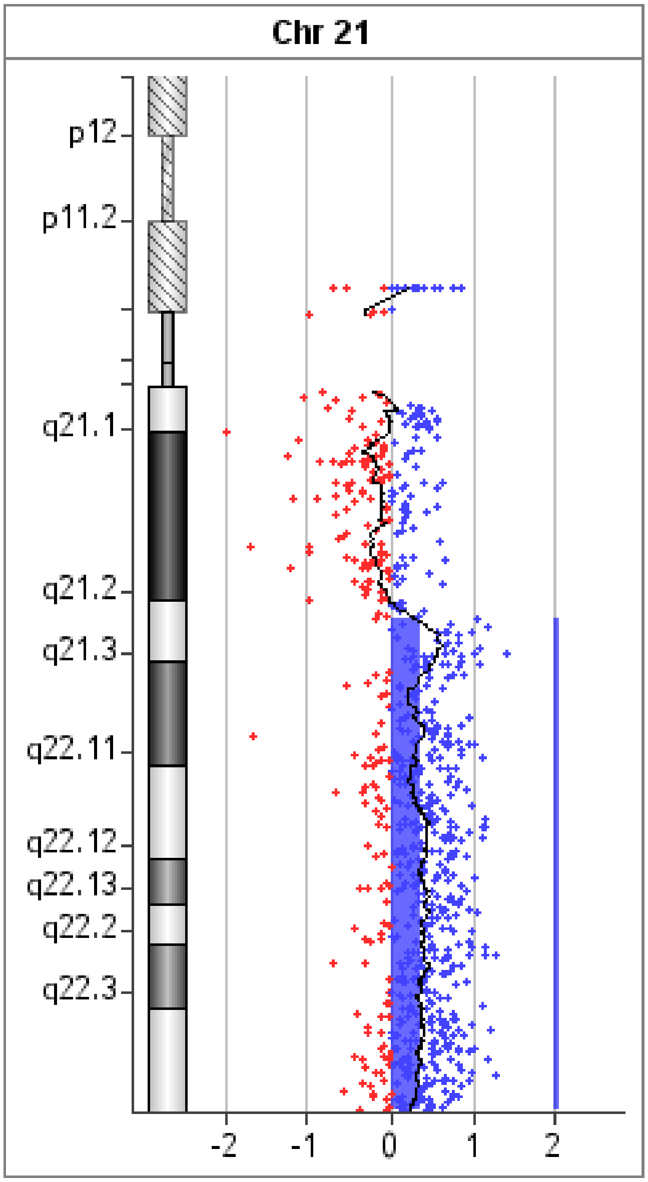

This is an intermediate report and not a final signed off report

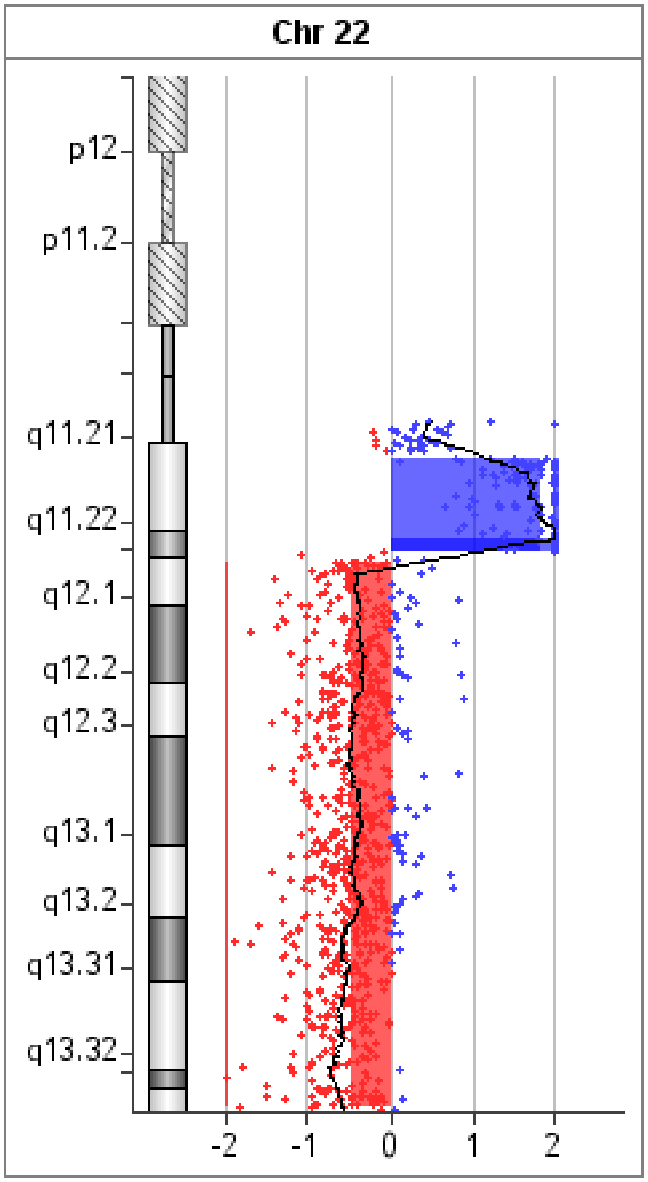

This is an intermediate report and not a final signed off report

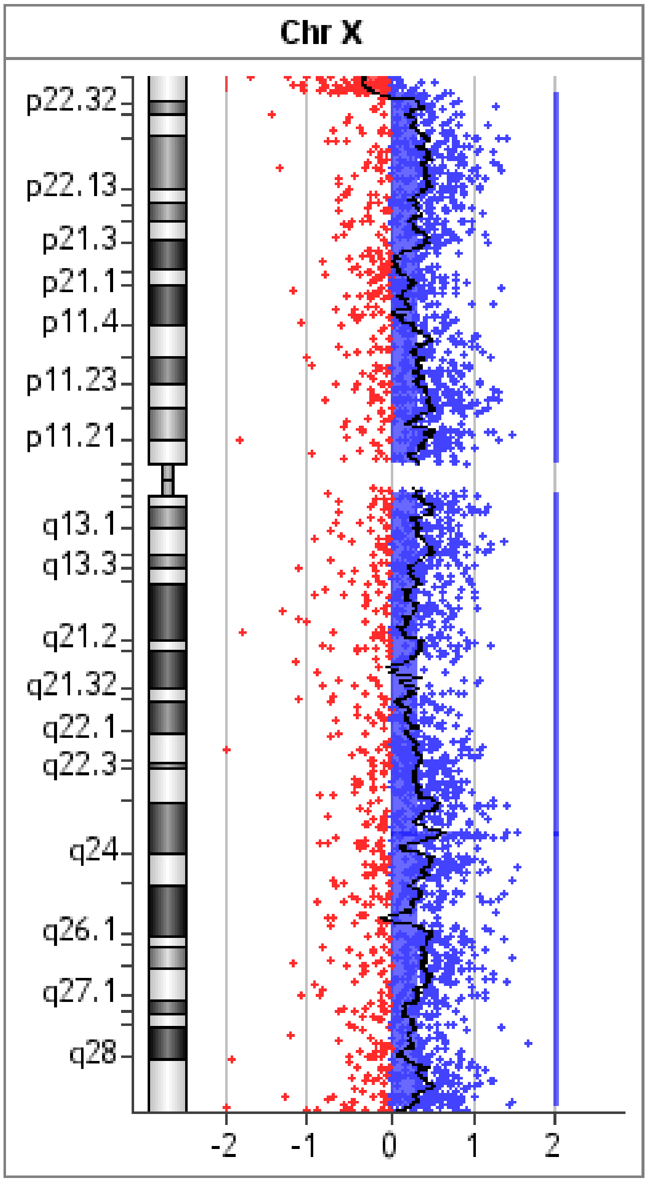

This is an intermediate report and not a final signed off report

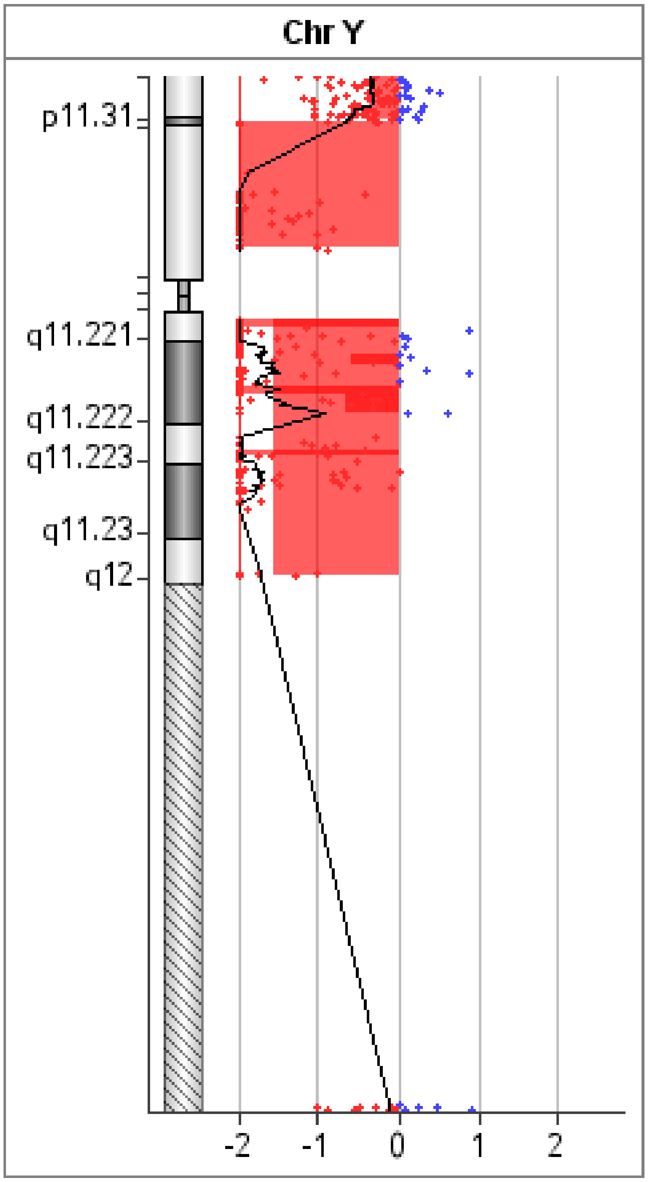

This is an intermediate report and not a final signed off report

Amp/Gain/Loss/Del Intervals Table

Chr	Start-Stop(bp)	Size(kb)	Cytoband	#Probes	Amp/Gain/Loss/Del	P-value	Annotations
chr1	852863-55252418	54,400	p36.33 - p32.3	1281	0.420767	4.90E-324	FLJ39609, SAMD11, NOC2L...
chr1	107867286-121281170	13,414	p13.3 - p11.2	305	0.396483	8.00E-78	NTNG1, VAV3, SLC25A24...
chr1	162299940-249151859	86,852	q23.3 - q44	1737	0.377019	4.90E-324	NOS1AP, MIR556, C1orf111...
chr2	157511681-188546067	31,034	q24.1 - q32.1	576	0.363323	9.49E-122	GALNT5, ERMN, CYTIP...
chr2	189068304-225781442	36,713	q32.1 - q36.2	733	-0.439867	3.82E-224	GULP1, MIR561, DIRC1...
chr3	48371494-84777471	36,406	p21.31 - p12.1	705	-0.516846	2.74E-298	FBXW12, PLXNB1, CCDC51...
chr3	59764652-60088412	324	p14.2	11	-1.620060	8.66E-23	FHIT
chr3	60629805-60728584	99	p14.2	3	-1.865281	4.53E-10	FHIT
chr3	93580736-197840339	104,260	q11.1 - q29	1900	-0.415205	4.90E-324	PROS1, ARL13B, STX19...
chr3	102332721-149307763	46,975	q12.3 - q25.1	866	-0.515204	1.47E-15	MIR548A3, ALCAM, CBLB...
chr3	155161736-187716648	32,555	q25.31 - q27.3	589	-0.280233	6.93E-19	PLCH1, C3orf33, SLC33A1...
chr4	28828344-37236650	8,408	p15.1 - p14	68	-0.299802	1.74E-11	PCDH7, ARAP2, DTHD1
chr4	41956926-47466088	5,509	p13 - p12	82	-0.250742	5.90E-10	TMEM33, DCAF4L1, SLC30A9...
chr4	160517264-163435909	2,919	q32.1 - q32.2	30	-1.715949	6.40E-129	FSTL5
chr5	151737-44268593	44,117	p15.33 - p12	630	0.396707	1.69E-158	PLEKHG4B, LRRC14B, CCDC127...
chr5	871638-14011580	13,140	p15.33 - p15.2	175	0.613636	5.32E-15	BRD9, TRIP13, NKD2...
chr5	144978575-145904094	926	q32	22	0.511440	5.20E-11	PRELID2, GRXCR2, SH3RF2...
chr5	163813707-164485709	672	q34	8	-0.905327	2.64E-12	
chr6	170426-16759504	16,589	p25.3 - p22.3	263	0.440910	4.92E-83	DUSP22, IRF4, EXOC2...
chr6	30271385-51732931	21,462	p22.1 - p12.3	582	0.538516	1.90E-268	HCG18, TRIM39, TRIM39-RPP21...
chr6	31548354-31826999	279	p21.33	31	0.987129	8.60E-12	LTB, LST1, NCR3...

This is an intermediate report and not a final signed off report

Chr	Start-Stop(bp)	Size(kb)	Cytoband	#Probes	Amp/Gain/Loss/Del	P-value	Annotations
chr6	38252496-51732931	13,480	p21.2 - p12.3	285	0.705417	1.59E-14	BTBD9, GLO1, DNAH8...
chr6	87429974-91598727	4,169	q14.3 - q15	90	0.366184	4.58E-21	HTR1E, CGA, ZNF292...
chr6	135238540-170890108	35,652	q23.3 - q27	627	0.381918	1.69E-146	ALDH8A1, HBS1L, MIR3662...
chr7	65972673-159088636	93,116	q11.21 - q36.3	2004	0.552672	4.90E-324	LOC493754, KCTD7, RABGEF1...
chr7	66096058-102114142	36,018	q11.21 - q22.1	697	0.314866	1.95E-64	KCTD7, RABGEF1, LOC729156...
chr7	104179715-159088636	54,909	q22.2 - q36.3	1265	0.681932	1.76E-35	LHFPL3, LOC645591, LOC723809...
chr8	83898547-89180109	5,282	q21.13 - q21.3	80	-0.263830	1.09E-10	RALYL, LRRCC1, E2F5...
chr8	130425357-130788501	363	q24.21	9	0.747999	7.56E-10	GSDMC
chr9	20778714-31510599	10,732	p21.3 - p21.1	152	-2.081331	4.90E-324	KIAA1797, PTPLAD2, IFNB1...
chr9	21036673-23690332	2,654	p21.3	51	-3.194464	1.36E-65	IFNB1, IFNW1, IFNA21...
chr9	23933298-24449398	516	p21.3	4	-3.744545	2.42E-12	
chr9	25583028-26465048	882	p21.3 - p21.2	8	-3.580202	1.42E-19	TUSC1
chr9	26649384-28519628	1,870	p21.2 - p21.1	37	-0.453681	4.00E-157	C9orf82, PLAA, IFT74...
chr9	28606661-29137041	530	p21.1	10	-3.609632	5.55E-24	LINGO2, MIR876, MIR873
chr9	30520898-31263003	742	p21.1	10	-3.121819	1.03E-13	
chr9	37129266-38403251	1,274	p13.2 - p13.1	31	0.593956	2.54E-19	ZCCHC7, GRHPR, ZBTB5...
chr9	71095721-94124443	23,029	q21.11 - q22.31	380	-0.541059	7.07E-177	PGM5, C9orf71, PIP5K1B...
chr9	130923464-131109445	186	q34.11	8	1.092968	4.08E-17	C9orf16, CIZ1, DNM1...
chr9	133654917-134139531	485	q34.12 - q34.13	18	2.292282	7.25E-151	ABL1, QRFP, FIBCD1...
chr10	87857715-135234843	47,377	q23.1 - q26.3	973	-0.502481	4.90E-324	GRID1, MIR346, WAPAL...
chr12	230421-23265912	23,035	p13.33 - p12.1	529	-0.488759	1.99E-199	IQSEC3, LOC574538, SLC6A12...
chr13	19296544-115011507	95,715	q11 - q34	1716	-0.453800	4.90E-324	LOC284232, LOC348021, PHF2P1...

This is an intermediate report and not a final signed off report

Chr	Start-Stop(bp)	Size(kb)	Cytoband	#Probes	Amp/Gain/Loss/Del	P-value	Annotations
chr13	23777913-74262909	50,485	q12.12 - q22.1	918	-0.548463	7.38E-15	SGCG, SACS, TNFRSF19...
chr13	81137986-81454116	316	q31.1	5	2.051843	8.33E-52	
chr13	90457836-93989566	3,532	q31.3	65	1.301068	5.60E-314	MIR622, LOC144776, MIR17HG...
chr13	90457836-92416896	1,959	q31.3	34	1.924095	6.29E-23	MIR622, LOC144776, MIR17HG...
chr13	92494334-92922405	428	q31.3	12	-0.757262	1.54E-76	GPC5
chr13	92973314-93309763	336	q31.3	9	2.159516	2.00E-12	GPC5
chr13	93390362-93780465	390	q31.3	6	-0.424447	2.36E-30	GPC5
chr13	93925051-93989566	65	q31.3	3	2.663395	1.04E-10	GPC6
chr14	20472548-107258824	86,786	q11.2 - q32.33	1727	-0.451063	4.90E-324	OR4K14, OR4K13, OR4L1...
chr15	20481702-26951736	6,470	q11.1 - q12	79	-0.274504	2.82E-11	HERC2P3, GOLGA6L6, GOLGA8C...
chr16	79245968-85744160	6,498	q23.2 - q24.1	145	0.392485	1.88E-37	WWOX, MAF, DYNLRB2...
chr17	148092-3962522	3,814	p13.3 - p13.2	121	-0.486275	2.49E-47	RPH3AL, C17orf97, FAM101B...
chr17	9739759-10049224	309	p13.1	16	-0.639248	2.83E-12	GLP2R, RCVRN, GAS7
chr17	10127678-12698956	2,571	p13.1 - p12	49	0.346861	3.08E-11	MYH13, MYH8, MYH4...
chr17	12892005-14013054	1,121	p12	19	-0.720345	3.53E-17	ARHGAP44, ELAC2, HS3ST3A1...
chr17	14654074-19959014	5,305	p12 - p11.2	130	0.368079	6.98E-30	CDRT7, PMP22, TEKT3...
chr17	20141913-22205821	2,064	p11.2 - p11.1	29	-0.611404	4.11E-19	SPECC1, CCDC144C, LGALS9B...
chr17	26877778-27926708	1,049	q11.2	46	0.504960	1.38E-20	UNC119, PIGS, ALDOC...
chr17	28206862-29385441	1,179	q11.2	30	-0.567062	5.48E-17	SSH2, EFCAB5, CCDC55...
chr18	142096-3100173	2,958	p11.32 - p11.31	58	0.415208	7.99E-18	USP14, THOC1, COLEC12...
chr18	8113468-10949636	2,836	p11.23 - p11.21	61	0.465438	5.91E-23	PTPRM, LOC100192426, RAB12...
chr18	19506073-28354953	8,849	q11.2 - q12.1	132	0.498759	4.78E-54	GATA6, CTAGE1, RBBP8...

This is an intermediate report and not a final signed off report

Chr	Start-Stop(bp)	Size(kb)	Cytoband	#Probes	Amp/Gain/Loss/Del	P-value	Annotations
chr18	39600614-77982126	38,382	q12.3 - q23	638	-0.554992	2.18E-311	PIK3C3, RIT2, SYT4...
chr20	309960-26187794	25,878	p13 - p11.1	484	-0.514838	6.88E-204	SOX12, NRSN2, TRIB3...
chr21	25203765-48018909	22,815	q21.2 - q22.3	545	0.364229	3.46E-116	NCRNA00158, MIR155HG, MIR155...
chr22	18919942-23627391	4,707	q11.21 - q11.23	125	1.827931	4.90E-324	PRODH, DGCR5, DGCR9...
chr22	22892487-23627391	735	q11.22 - q11.23	21	2.401546	7.24E-13	PRAME, LOC648691, POM121L1P...
chr22	24092647-51104602	27,012	q11.23 - q13.33	732	-0.472160	1.75E-259	ZNF70, VPREB3, C22orf15...
chrX	61091-2502925	2,442	p22.33	88	-0.355334	1.58E-19	PLCXD1, GTPBP6, NCRNA00107...
chrX	2709027-58051765	55,343	p22.33 - p11.21	989	0.332787	3.29E-172	XG, GYG2, ARSD...
chrX	62645308-154754171	92,109	q11.1 - q28	1575	0.335781	4.22E-278	LOC92249, ARHGEF9, MIR1468...
chrX	113275135-114305500	1,030	q23	22	0.857009	2.28E-11	HTR2C, SNORA35, MIR764...
chrY	11091-2452925	2,442	p11.32	88	-0.355334	1.58E-19	PLCXD1, GTPBP6, NCRNA00107...
chrY	2656461-9901314	7,245	p11.31 - p11.2	59	-2.299345	3.16E-322	RPS4Y1, ZFY, TGIF2LY...
chrY	14061053-28767604	14,707	q11.21 - q11.23	144	-1.551901	4.90E-324	GYG2P1, TTTY15, USP9Y...
chrY	14061053-14463950	403	q11.21	7	-3.411399	1.01E-22	
chrY	16017052-16640171	623	q11.221	9	-0.591284	9.18E-14	VCY1B, VCY, NLGN4Y
chrY	17890214-18225843	336	q11.221	4	-3.092707	2.48E-11	
chrY	18255453-19291733	1,036	q11.221	11	-0.644042	4.10E-16	
chrY	21491899-21768349	276	q11.222	5	-3.211339	6.10E-13	BCORP1, CYorf15A, CYorf15B

Amp=Amplification Del=Deletion

Total Amp/Gain/Loss/Del Intervals: 83

This is an intermediate report and not a final signed off report

Analysis Settings

Design	:	021924_20101001	Sample Name	:	252192435816_2_2
Genome	:	hg19	Aberration Algorithm	:	ADM-2
Threshold	:	6.0	Fuzzy Zero	:	OFF
GC Correction	:	ON	Window Size	:	2Kb
Centralization (legacy)	:	OFF	Diploid Peak Centralization	:	ON
SNP Copy Number	:	OFF	LOH	:	OFF
Combine Replicates (Intra Array)	:	ON	Array Level Filter	:	NONE
Metric Set Filter	:	NONE	Aberration Filter	:	Minimum Number of Probes for Amplification >= 3 AND Nesting Level <= 100 AND Minimum Avg. Absolute Log Ratio for Amplification >= 0.25 AND Minimum Size (Kb) of Region for Amplification >= 0.0 AND Minimum Size (Kb) of Region for Deletion >= 0.0 AND Minimum Number of Probes for Deletion >= 3 AND Minimum Avg. Absolute Log Ratio for Deletion >= 0.25 AND Minimum Number of Probes for Gain >= 3 AND Minimum Number of Probes for Loss >= 3 AND Minimum Avg. Absolute Log Ratio for Gain >= 0.25 AND Minimum Avg. Absolute Log Ratio for Loss >= 0.25 AND Minimum Size (Kb) of Region for Gain >= 0.0 AND Minimum Size (Kb) of Region for Loss >= 0.0
Feature Level Filter	:	gIsSaturated = true OR rIsSaturated = true OR gIsFeatNonUnifOL = true OR rIsFeatNonUnifOL = true OR LogRatio = 0; Include matching values=false	Design Level Filter	:	Homology = 0 OR IsPseudoautosomal = 1
LOH Filter	:	NONE	Genomic Boundary	:	OFF
Show Flat Intervals	:	false	Template Name	:	Default Cyto Report Template - CGH

This is an intermediate report and not a final signed off report

Notes

Sample Notes

No notes available.

Amp/Gain/Loss/Del Interval Notes

No notes available.

This is an intermediate report and not a final signed off report
